# Characterisation of intracellular molecular mechanisms modulated by carnosine in porcine myoblasts under basal and oxidative stress conditions

**DOI:** 10.1371/journal.pone.0239496

**Published:** 2020-09-18

**Authors:** Marie-France Palin, Jérôme Lapointe, Claude Gariépy, Danièle Beaudry, Claudia Kalbe

**Affiliations:** 1 Agriculture and Agri-Food Canada, Sherbrooke Research and Development Centre, Sherbrooke, Québec, Canada; 2 Agriculture and Agri-Food Canada, Saint-Hyacinthe Research and Development Centre, Saint-Hyacinthe, Québec, Canada; 3 Leibniz Institute for Farm Animal Biology, Institute of Muscle Biology and Growth, Dummerstorf, Germany; Tohoku University, JAPAN

## Abstract

Carnosine is a naturally occurring histidine-containing dipeptide present at high concentration in mammalian skeletal muscles. Carnosine was shown to affect muscle contraction, prevent the accumulation of oxidative metabolism by-products and act as an intracellular proton buffer maintaining the muscle acid-base balance. The present study was undertaken to gain additional knowledge about the intracellular mechanisms activated by carnosine in porcine myoblast cells under basal and oxidative stress conditions. Satellite cells were isolated from the skeletal muscles of 3 to 4 day-old piglets to study the effect of 0, 10, 25 and 50 mM carnosine pre-treatments in cells that were exposed (0.3 mM H_2_O_2_) or not to an H_2_O_2_-induced oxidative stress. Study results demonstrated that carnosine acts differently in myoblasts under oxidative stress and in basal conditions, the only exception being with the reduction of reactive oxygen species and protein carbonyls observed in both experimental conditions with carnosine pre-treatment. In oxidative stress conditions, carnosine pre-treatment increased the mRNA abundance of the nuclear factor, erythroid 2 like 2 (*NEF2L2*) transcription factor and several of its downstream genes known to reduce H_2_O_2_. Carnosine prevented the H_2_O_2_-mediated activation of p38 MAPK in oxidative stress conditions, whereas it triggered the activation of mTOR under basal conditions. Current results support the protective effect of carnosine against oxidative damage in porcine myoblast cells, an effect that would be mediated through the p38 MAPK intracellular signaling pathway. The activation of the mTOR signaling pathway under basal condition also suggest a role for carnosine in myoblasts proliferation, growth and survival.

## Introduction

Carnosine (β-alanyl-L-histidine) and its methylated analogs, anserine and ophidine, are naturally occurring histidine-containing dipeptides (HCDs) present in various mammalian tissues with the highest concentrations found in the skeletal muscle, heart and brain [1]. Carnosine exhibits multiple biochemical activities including intracellular pH buffering, antioxidant, carbonyl scavenging and metal ion chelating properties [1]. Carnosine also prevents the formation of advanced glycoxidation and lipoxidation end-products known to increase with the aging process and to accumulate in chronic diseases such as diabetes, atherosclerosis and Alzheimer [1]. Given the multiple biochemical functions of carnosine, its therapeutic potential has been investigated in a number of studies. For instance, it was demonstrated that carnosine can protect against diabetic complications in experimental rat models of diabetic retinopathy and nephropathy [2, 3]. Carnosine can also inhibit tumor proliferation in athymic nude mice transplanted with human colon cancer cells [4], show protective effects in a mice model of Alzheimer’s disease [5] and may increase efficiency of DOPA therapy in Parkinson’s disease [6].

The amount of carnosine found in skeletal muscle represents 99% of the HCDs of an organisms [1], thus suggesting an important role for carnosine in this tissue. Dutka et al. [7] indeed demonstrated that physiological concentrations of carnosine can increase the Ca^2+^ sensitivity and promote Ca^2+^ release in isolated human muscle fibers. In addition, a positive ergogenic effect of β-alanine supplementation was found on certain types of exercises, with greater effects observed in non-trained individuals [8]. Carnosine is also known to have a role in the maintenance of the pH homeostasis, thus reducing muscle fatigue [9, 10], and to prevent the accumulation of oxidative metabolism by-products [1]. Moreover, Rezzani et al. [11] recently reported that carnosine, anserine and carnosinol (a carnosine analog) can reduce oxidative stress, apoptosis and inflammation in rat L6 myotubes.

Two recent papers have attracted the interest of the swine industry on the advantages of increasing muscle carnosine to improve pigs growing performances and meat quality. In the first paper, dietary supplementation of carnosine was found to increase pigs average daily gain, average daily feed intake and live weight [12] and, in the second paper, pigs with high muscle carnosine content had improved water holding capacity and meat color values [13]. The oxidative status of pigs skeletal muscle can greatly impact meat quality and carnosine was recently reported to improve the antioxidant capacity in skeletal muscles of pigs supplemented with carnosine [14]. However, since muscle carnosine content was not quantified in that paper, it remains unclear whether the observed effects are really due to an increase of muscle carnosine. The importance of satellite/myoblast cells proliferation and fusion in supporting hypertrophy of existing muscle fibers during postnatal growth was demonstrated recently [15] as well as their key roles in muscle regeneration [16]. The carnosine ability to improve pig growing performances [12] could be explained, at least in part, by its effect on myoblasts replicative capacity. This was indeed demonstrated by Maier et al. [17] reporting an increase in human myoblasts proliferation after addition of carnosine, 30 days upon establishment of cell cultures.

Collectively, the abovementioned studies demonstrate that carnosine has an important role in skeletal muscle growth and function, however the intracellular molecular mechanisms modulated by carnosine need further characterization. The present study was therefore undertaken to gain additional knowledge about the intracellular mechanisms activated by carnosine in porcine satellite cell-derived myoblasts under basal and oxidative stress conditions. More specifically, our primary objectives were to investigate the effect of carnosine on 1) myoblasts viability and proliferation, 2) oxidative stress biomarkers, 3) antioxidant enzyme activities and gene expression and, 4) mitochondrial bioenergetics. In addition, the effect of carnosine on the activation of mitogen-activated protein kinases (MAPK), responsive to various stress stimuli, and of the mammalian target of rapamycin (mTOR), known to control cell proliferation, growth and survival, was also assessed.

## Materials and methods

### Isolation of porcine muscle satellite cells

Satellite cells were isolated from the skeletal muscles of 3 to 4-day old crossbred male piglets (n = 34) using a Percoll gradient method as previously described in Metzger et al. [18]. This method was chosen because it provides highly pure satellite cells in rats (> 90%) [19] and pigs (>95%) [18]. Moreover, satellite cells isolated with this method conserve their myogenic phenotype, with expression of paired Box 7 (*PAX7*) in satellite cells and expression of myogenic differentiation 1 (*MYOD*) and myogenin (*MYOG*) genes after 72 hours of proliferation [18]. In addition, these cells are also able to differentiate with relatively high fusion index. The *longissimus dorsi* muscles from 2 to 3 piglets were used for each isolation procedure. A single pool of primary muscle cells was established from all isolation procedures and aliquots were kept in a -150°C ultra low freezer. Primary cell pooling is known to reduce biological variability of cell donors. It also offers the possibility to conduct long-term projects with multiple experiments and replications, which was the case for the current study. In a recent paper by Metzger et al. [18] it was clearly demonstrated that cell pools can be used as a relevant method to reflect the cellular behavior of unpooled cells. The percentage of myogenic cells was determined as described in Mau et al. [20] using a BD FACSCanto II flow cytometer (BD Biosciences, San Jose, CA, USA). For this analysis, a mouse monoclonal anti-desmin antibody from pig stomach (dilution 1:80; mAb DE-U-10, ascite fluids; D1033 Sigma-Aldrich, Oakville, ON, Canada) and a fluorescein-conjugated horse anti-mouse IgG secondary antibody (dilution 1:100; FI-2000 Vector Laboratories, Burlingame, CA, USA) were used. The cell pool contained >95% of desmin-positive cells. Cell viability was >97% as determined with a Countness Automated Cell Counter (Life technologies, Burlington, ON, Canada). Animal husbandry and slaughter were conducted according to the recommended Canadian code of practice [21] and approved by the Institutional Animal Care Committee of the Sherbrooke Research and Development Centre of Agriculture and Agri-Food Canada.

### Cell culture and treatments

**Fig 1:**
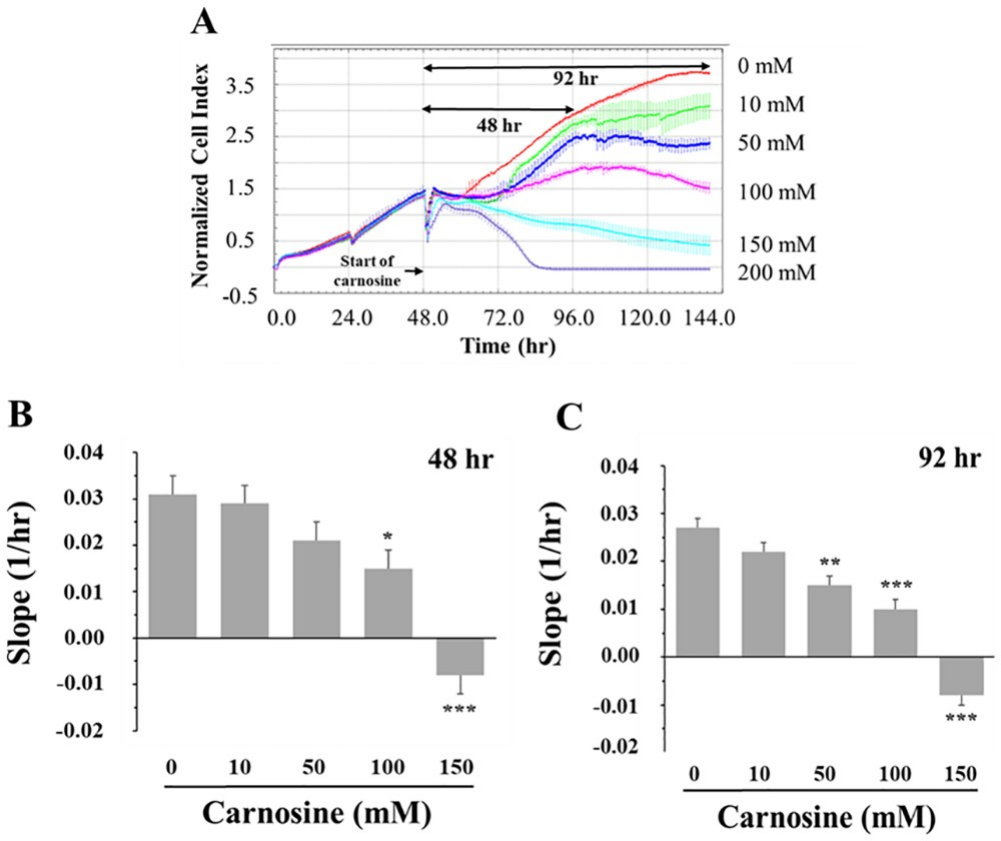
Effect of carnosine concentration on myoblast proliferation using real-time cell impedance monitoring. (A) Representative cell growth kinetic curves monitored by the xCELLigence system. After 48 h in growth medium, the cells were treated with carnosine (0, 10, 50, 100, 150 and 200 mM) for 92 h. Impedance measurements were recorded every 30 min and are expressed as normalized cell index (nCI). For each carnosine concentration, four wells (n = 4) were used. The rate of cell proliferative growth was determined by calculating the slope (1/hr) between 48 and 96 hours (B) and between 48 and 140 hours (C). Values correspond to the means ± SEM of n = 3 independent experiments. SEM between 48 and 96 hours = 0.004 (B), SEM between 48 and 140 hours = 0.002 (C). Each carnosine treatment was compared to the control (0 mM carnosine) using a Dunnett correction. *P≤0.05; **P≤0.01; ***P≤0.001.

Aliquots of pooled myogenic cells were thawed and seeded in gelatin-coated 6 (1.5 x 10^5^ cells/well) or 96 (5 x 10^3^ cells/well) well plates. Cells were grown in Growth Medium (GM: DMEM containing 10% fetal bovine serum, 10% horse serum, 1% glutamine, 1% penicillin/streptomycin and 1% amphotericin B; Sigma-Aldrich) for 48 h and then pre-treated with carnosine (0, 10, 25 and 50 mM; Sigma-Aldrich C9625) for another 48 h. The 10 and 25 mM carnosine concentrations correspond to observed physiological range for pig skeletal muscles [1] and the 50 mM carnosine represents a supra-physiologic concentration. Cell exposure time with carnosine was determined based on results with real-time cell impedance monitoring (Fig 1) indicating similar cell proliferative growth up to 48 h for the 0, 10 and 50 mM doses. These cells were then either treated with 0.3 mM hydrogen peroxide (H_2_O_2_, 1 h; Sigma-Aldrich H1009) to induce an oxidative stress or without H_2_O_2_ (1 h; basal conditions) before performing analyses described below. Hydrogen peroxide concentration and exposure time were determined based on the CellTiter 96^®^ AQueous One Solution Cell Proliferation Assay (Promega, Madisson, Wi, USA), as described below.

### Cell proliferation and viability analyses

The effect of carnosine on porcine myoblasts proliferative growth was first analyzed with real-time cell impedance monitoring using an xCELLigence RTCA SP system (ACEA Biosciences Inc., San Diego, CA, USA). Cells were grown in GM in gelatin-coated 96-well culture plates (5 x 10^3^/well) having microelectrodes in the bottom of each well (e-plates 96, ACEA Biosciences Inc.). Impedance measurements were recorded every 30 min for up to 140 h and results were expressed as normalized cell index (nCI). After 48 h, the cells were treated with carnosine (0, 10, 50, 100, 150 and 200 mM) up to 96 h. Three (n = 3) independent experiments were performed with four repetitions of each carnosine concentration and cell proliferative growth was determined by calculating the mean change of nCI over different period of time (slope; 1/hr). The nCI slope values provided by the system (Software RTCA, Version 1.2.1.) between 48 and 96 hours and between 48 and 140 hours were used for statistical analyses. This analysis allowed us to eliminate the highest carnosine concentrations (100, 150 and 200 mM) based on the observed negative impact on cell proliferative growth (see Results section).

Quantification of cell proliferation was also monitored by measuring the incorporation of BrdU into newly synthesized DNA of proliferating myoblasts. For this analysis, a colorimetric immunoassay (Cell Proliferation ELISA BrdU, colorometric; Roche Applied Science) was used according to the manufacturer’s instructions. In brief, cell treatments were as described above and myoblasts were then incubated for an additional 5 h with 10 μM of the BrdU labeling solution. The labeling medium was discarded by suction and the cells incubated for 2 h with the anti-BrdU-POD solution. The immune complex was detected at 450 nm after addition of the substrate solution. For these analyses, the H_2_S0_4_ (1 M) stop solution option was selected. For each treatment, n = 4 independent experiments were performed in triplicate.

Cell viability was measured with the CellTiter 96^®^ AQueous One Solution Cell Proliferation Assay (Promega). This assay is based on the reduction of a 3-(4,5-dimethyl-thiazol-2-yl)-5-(3-carboxymethoxyphenyl)-2-(4-sulfophenyl)-2H-tetrazolium (MTS) compound by metabolically active cells to generate a colored product that is detected at 490 nm. In this study, the MTS assay is used as a surrogate of cell viability measurements. For the first MTS assay, cells were seeded in gelatin-coated 96-well plates and incubated for 48 h. Cells were then exposed to different concentrations of H_2_O_2_ (0, 0.1, 0.2, 0.3, 0.5 and 1.0 μM) for 30 min, 1 h and 2 h. The CellTiter 96^®^ AQueous One solution reagent was added directly in each well and incubated for 5 h. Absorbance was measured at 480 nm using a Spectramax plus plate reader (Molecular Devices; San Jose, CA, USA). This first test enabled us to determine the H_2_O_2_ concentration (0.3 mM, 1 h) to be used in all induced oxidative stress experiments. The MTS assay was also performed for cells that were treated with carnosine (0, 10, 25 and 50 mM) and were exposed or not to H_2_O_2_ (0.3 μM, 1 h). Results are presented as mean absorbance values of triplicates from four independent experiments.

### Quantification of intracellular reactive oxygen species

The accumulation of intracellular reactive oxygen species (ROS) was measured with the Oxiselect^™^ Intracellular ROS Assay kit–Green Fluorescence (STA-342; Cell Biolabs, San Diego, CA, USA). This assay measures hydroxyl, peroxyl and other reactive oxygen species activity. Cells were seeded in black cell culture fluorometric 96-well plates and grown as described above for 48 h, followed by an additional 48 h with treatments. Quantification of intracellular ROS was then carried out according to the manufacturer’s instructions, the level of ROS being proportional to the amount of fluorescent 2’, 7’-dichlorodihydrofluorescein detected at 480 nm excitation and 530 nm emission. Assays were performed in duplicate for n = 4 independent experiments. Data are presented as the mean value of relative fluorescence unit (RFU).

### Determination of oxidative damage to proteins

Oxidative damage to proteins was quantified using the OxiSelectTM Protein Carbonyl ELISA Kit (STA-310; Cell Biolabs). Cells were grown in 6-well plates and treatments applied as described above. At the end of the treatment period, the GM was discarded and replaced by 500 μL of phosphate buffer (50 mM, pH 7.0) containing 1 mM EDTA. Cells were then collected by gently scraping the bottom of each well and samples were frozen overnight (-80°C). After thawing, cells were lysed by sonication with a Q125 SONICATOR (Qsonicata L.L.C.; Newtown, CT, USA) using 40% power output and 20 sec ON/10 sec OFF/20 sec ON pulses. A centrifugation at 10,000 x g for 15 min at 4°C was then performed to get rid of cell debris. The supernatant was collected and directly essayed for protein carbonyls according to the manufacturer’s protocol. An aliquot (15 μL) of the supernatant was kept to determine the protein concentration in each sample using a Bio-Rad Protein Assay (BIO-RAD, Mississauga, ON, Canada). Data are presented as mean values of n = 4 experiments assayed in duplicate and are expressed as nmol protein carbonyl/mg proteins.

### Superoxide dismutase and glutathione peroxidase activities

To measure the superoxide dismutase (SOD) enzyme activity, the cells were seeded in 6-well plates. At the end of the treatment period, the GM was replaced with 500 μL of cold HEPES buffer (20 mM, pH 7.2) containing 1 mM EGTA, 210 mM mannitol and 70 mM sucrose and cells were collected by gentle scraping. Cells were frozen and sonicated as described above. Cell lysates were then centrifuged at 1,500 x g for 5 min at 4°C and supernatants collected to perform the SOD activity assay using a Superoxide Dismutase Assay Kit (No. 706002; Cayman Chemical, Ann Harbor, MI, USA), which measures the activities of Cu/Zn, Mn and Fe SODs. The total protein concentration was also measured in each sample using the Bio-Rad Protein Assay (BIO-RAD). Data are presented as the mean values of n = 4 experiments (in duplicate) and are expressed as Unit/mg proteins. One unit corresponds to the amount of SOD needed to exhibit 50% dismutation of the superoxide radical.

The glutathione peroxidase (GPX) activity was determined using a spectrophotometric method adapted from Gunzler and Flohé [22]. The culture media was collected and discarded. 250 μL of potassium phosphate buffer (100 mM, pH 7.8), containing 0.5 M EDTA and protease inhibitors (leupeptin and phenylmethylsulfonyl fluoride (PMSF)), was added to each well (6-well plates) and cells were recovered by gentle scraping. Cell suspensions from two wells were pooled and sonicated as described above. Cell lysates were then centrifuged at 10,000 x g for 10 min (4°C) and the supernatant used for GPX activity determination. This assay is based on the reduction of cumene hydroperoxide (Sigma-Aldrich) by GPX while converting reduced glutathione to oxidized glutathione. Glutathione reductase then reduces oxidized glutathione to reduced glutathione with consumption of NADPH. The decrease of NADPH is measured at 340 nm and is proportional to the GPX activity. Four independent experiments were conducted and assays performed in triplicate. Activity values are reported as milliunit (mU) per mg of proteins, with 1 unit corresponding to 0.5 μM of oxidized NADPH per minute.

### Gene expression analyses

Total RNA was extracted from treated myoblasts (6-well plates) using QIAshredder columns (Qiagen; Toronto, ON, Canada), followed by RNeasy^®^ Fibrous Tissue Mini columns (Qiagen), which included a DNAse I digestion step directly on columns. For each treatment, the cells from two wells were pooled before performing the RNA extraction. Concentration and purity of extracted RNA was assessed with a NanoDrop Spectophotometer ND-1000 (NanoDrop Thechnologies, Wilmington, DE, USA) and a 1% agarose gel electrophoresis. Complementary DNA (cDNA) synthesis was carried out with the Superscript IV Reverse Transcriptase (200U/mL; Thermo Fisher Scientific, Waltham, MA, USA) and oligo(dT) 20 primers.

The relative mRNA abundance of genes involved in cellular defence against oxidative stress (Table 1) was quantified using real-time PCR analyses. Amplifications were performed in a 10-μL reaction volume containing forward and reverse primers at indicated concentrations (Table 1), 5 μL of 2x Power SYBR^®^Green Master Mix (PE Applied Biosystems; Foster City, CA, USA), 3 μL of 1:30 diluted cDNA and 0.05 μL of AmpErase Uracil N-Glycosylase (PE Applied Biosystems). Amplifications were done in triplicate using an ABI 7500 Fast Real-Time PCR System (PE Applied Biosystems) with the following cycling conditions: 2 min at 50°C AmpErase activation, followed by 10 min at 95°C and 40 cycles of 15 sec at 95°C (denaturation) and 45 sec at 60°C (annealing and polymerisation). In each 96-well plate, a standard curve was established in duplicate and made of serial dilutions of cDNA pools as previously described [23]. Selection of reference genes (RG) that were the least affected by treatments was carried out with the NormFinder algorithm [24]. The combination of *Actin Beta* (*ACTB*), *Beta-2-Microglobulin* (*B2M*) and *Ribosomal Protein Lateral Stalk Subunit P0* (*PRLP0*) RG was chosen as the normalization factor used for carnosine treatments without H_2_O_2_ (basal condition). For treatments with both carnosine and H_2_O_2_, a combination of *B2M*, *Hypoxanthine Phosphoribosyltransferase 1* (*HPRT1*) and *Ribosomal Protein L13a* (*RPLP13A*) RG was selected as the normalization factor. Relative quantity values were then obtained by dividing the relative quantity units of selected genes by those of the normalization factors. Statistical analyses were carried out using mean values from n = 4 independent experiments done in triplicate.

**Table 1:** Primer sequences used for real-time PCR amplifications of studied and reference genes in porcine myoblast cells.

Genes^1^	Primer sequence (5’→ 3’)^2^	GenBank accession no.	Primer (nM)	Ampl. Eff. (%)^3^
**Studied genes**				
*CAT*	(F) ACGTGCAGCGCTTCAACAG (R) CTCTCCTCCTCATTCAGTACGTTCA	NM_214301	300 300	97.5
*GPX1*	(F) CGATGCCACTGCCCTCAT (R) GGCCCACCAGGAACTTCTC	NM_214201	300 300	97.40
*GPX3*	(F) CTCGGAGATTCTGTCCACTCTCA (R) CCGTTCACGTCCCCTTTCT	NM_001115155	300 300	98.60
*GPX4*	(F) ACGGGCACATGGTGAACCT (R) ACCTCCGTCTTGCCTCATTG	NM_214407	300 300	101.40
*GSR*	(F) GGACAGTGGGACTCACAGAAG (R) CCTCTTTGTTGGCACACACC	XM_003133500	800 800	97.90
*NEF2L2*	(F) CAATGAGGTTTCTTCGGCTACA (R)TCGTCTACCTCAGTAGTGGCTAACTG	XM_003133500	300 300	102.58
*PRDX1*	(F) TCAGGCCTTCCAGTTCACTGA (R) AATATTCTTTGCTCTTCTGGACATCA	XM_003128042	300 300	102.30
*PRDX3*	(F) GCCATCTGGCTTGGATAAATACA (R) CGGGAAATTTGCTTGGTCAA	NM_001244531	300 300	103.10
*PRDX4*	(F) TCAAGCATTCCAGTACACTGACAA (R) AGCTGGATCTGGGATTATAGTTTCA	XM_001927369	300 300	99.40
*SOD1*	(F)CACTGTGTACATCGAAGATTCTGTGA (R) GCCCAAGTCATCTGGTTTTTCA	NM_001190422	300 300	99.30
*SOD2*	(F) TCTGGACAAATCTGAGCCCTAAC (R) CGGATACAGCGGTCAACTTCTC	NM_214127	300 300	102.50
*SOD3*	(F) ATGCTGACGCTGCTCTGTGCTTA (R) TCCTGCCAGATCTCCGTCACTTT	NM_001078688	800 800	102.50
*TXNRD1*	(F) GGCCACTAACAGTGACGAAACC (R) TGCTCTTCATCCGTCACAGGTA	NM_214154	800 800	100.27
*TXNRD2*	(F) GATGCGTCCCAGTGTTACATAAAG (R) CTTGAGTGCCAGAGCAAATCC	XM_003133500	800 800	102.58
**Reference genes**				
*ACTB*	(F) CATCACCATCGGCAACGA (R) GGATGTCGACGTCGCACTT	XM_003124280	300 300	97.20
*B2M*	(F) CTTTCAGCAAGGACTGGTCTTTCTAC (R) GTGGTCTCGATCCCACTTAACTATC	NM_213978.1	300 300	99.70
*HPRT1*	(F) GACCAGACTTTGTTGGATTTGAAA (R) CAAACATGATTCAAGTCCCTGAAG	NM_001032376	300 300	96.70
*PRLP0*	(F) TGCTTGACATCACCGAGGAA (R) TTGGGTAACCAATCTGCAGACA	NM_001098598	300 300	97.80
*PRLP13A*	(F) GTGGCCAAGCAGGTACTTCTG (R) GGGACGGGTTGGTATTCATG	NM_001244068	300 300	101.10

^1^ *ACTB*, Actin Beta; *B2M*, Beta-2-Microglobulin; *CAT*, Catalase; *GPX1*, Glutathione Peroxidase 1; *GPX3*, Glutathione Peroxidase 3; *GPX4*, Glutathione Peroxidase 4; *GSR*, Glutathione-Disulfide Reductase; *HPRT1*, Hypoxanthine Phosphoribosyltransferase 1; *NEF2L2*, Nuclear Factor, Erythroid 2 Like 2; *PRDX1*, Peroxiredoxin 1; *PRDX3*, Peroxiredoxin 3; *PRDX4*, Peroxiredoxin 4; *PRLP0*, Ribosomal Protein Lateral Stalk Subunit P0; *RPLP13A*, Ribosomal Protein L13a; *SOD1*, Superoxide Dismutase 1; *SOD2*, Superoxide Dismutase 2; *SOD3*, Superoxide Dismutase 3; *TXNRD1*, Thioredoxin Reductase 1; *TXNRD2*, Thioredoxin Reductase 2.

^2^F = forward and R = reverse primers.

^3^Amplification efficiency (E) was calculated with E = 10[−1/slope].

### Western Blot analyses

After all treatments (6-well plates), myoblast cells were washed with Dulbecco’s Phosphate-Buffered Saline (DPBS) and then lysed with 0.3 mL of M-PER Mammalian Protein Extraction Reagent (#78501; Thermo Fisher Scientific) containing protease (HALT^™^ Protease Inhibitor Cocktail, EDTA-Free; Thermo Fisher Scientific) and phosphatase (HALT^™^ Phosphatase Inhibitor Cocktail; Thermo Fisher Scientific) inhibitors. After 10 min of incubation, the cells from 3 wells were pooled and a sonication was performed as described above. The cell lysate was then centrifuged at 14,000 x g for 10 min at 4°C and the supernatant collected for Western Blot analyses. Protein concentration was determined with the Bio-Rad Protein Assay (BIO-RAD). Equal amounts of protein (10 μg) were loaded in each well of a 10% SDS-polyacrylamide gel and separated by electrophoresis (SDS-PAGE). Proteins were then transferred to nitrocellulose membranes that were blocked with 5% skimmed milk in TBST (20 mM Tris-HCl, pH 7.5, 150 mM NaCl, 0.1% tween 20) for 2 h. Membranes were then incubated overnight at 4°C with primary antibodies against p44/42 MAPK (Erk1/2) (1:2000; #4695S), phospho-p44/42 MAPK at Thr202/Tyr204 (1:1000; #4370S), p38 MAPK (1:2000; #9212S), phospho-p38 MAPK at Thr180/Tyr182 (1:1000; #4511S), SAPK/JNK (1:2000; #9252S), phospho-SAPK/JNK at Thr183/Tyr185 (1:1000; #9251S), mTOR (7C10) (1:10000; #2983S), phospho-mTOR at Ser2448 (1:1000; #2971S), p70 S6 Kinase (1:1000; #9202S), phospho-p70 S6 Kinase at Thr389 (1:1000; #9205S), 4E-BP1 (1:10000; #9452S), phospho-4E-BP1 at Thr70 (1:1000; #9455S), followed by a 1 h incubation with an anti-rabbit IgG, horseradish peroxidase (HRP)-linked secondary antibody (1:5000; # 7074S). These antibodies were from Cell Signaling Technology (Beverly, MA, USA). Mouse monoclonal anti-α-Tubulin primary antibodies (1:10000; # T-5168; Sigma-Aldrich) and anti-mouse IgG HRP-linked secondary antibodies (1:10000; # NA931; GE Healthcare Life Sciences, Piscataway, NJ, USA) were used as loading control. The p44/42 MAPK-inhibitor U0126 (#19–147; Sigma-Aldrich), the p38 MAPK-inhibitor SB 203580 (#S 8307; Sigma-Aldrich) and the pSAPK/JNK-inhibitor SP600125 (#S5567; Sigma-Aldrich) were used to verify the specificity of phosphorylated signals. Protein detection was performed by enhanced chemiluminescence (ECL Prime Western Blotting Detection Reagent; GE Healthcare Life Sciences) and signal quantification with a Fusion FX5 fluorescence and chemiluminescence imaging system (Montreal Biotech, Dorval, QC, Canada). Phosphorylated and total proteins were first normalized with corresponding α-Tubulin data. These values were then used to present data as a ratio of phosphorylated protein/total protein (e.g. p-p38/p38). Data are presented as the mean value of n = 4 independent experiments.

### Measurements of cellular oxygen consumption rate

Cellular oxygen consumption rate analyses were performed using the Agilent Technologies Seahorse XFp analyzer (8 chambers; Seahorse Bioscience, North Billerica, MA, USA) and the Agilent Seahorse XFp Cell Mito Stress Test Kit. On day 1 of the assay, myoblasts were seeded in GM (4 x 10^3^ cells/chamber) in six chambers of the Seahorse XFp Cell Culture Miniplate. The two remaining chambers were kept as negative controls (no cells). Cells were grown in GM at 37°C and 6% CO_2_ in a humidified incubator. The culture medium was removed on day 2 and replaced with fresh GM (with or without carnosine). After completion of carnosine and H_2_O_2_ treatment periods (at 90% confluence), the GM was removed (leaving 20 μL of GM to avoid cell dryness) and replaced with 200 μL of assay medium (HEPES base medium (5 mM) containing 1 mM sodium pyruvate, 2 mM glutamine and 25 mM glucose; pH 7.4). The cell culture miniplates were incubated 1 h, at 37°C and without CO_2_. Components of the Cell Mito Stress Test Kit were then used to assess oxygen consumption rate. Basal respiration was first measured, followed by sequential injection of modulators of components of the mitochondrial electron transport chain (oligomycin (1 μM), carbonyl cyanide-4 (trifluoromethoxy) phenylhydrazone (FCCP; 2 μM) and a mixture of rotenone and antimycin A (0.5 μM)). At the end of each assay, cells were collected to determine total cellular proteins. Data were analysed using the Seahorse Wave Desktop Software and are expressed as oxygen consumption rate (OCR; pmol/min/μg protein). The mean values of 5 independent experiments were used for statistical analyses. These analyses allowed for the determination of the effect of treatments on basal respiration, ATP-linked respiration, maximal respiration, spare respiratory capacity, proton (H+) leak and non-mitochondrial respiration.

### Statistical analyses

All data were analysed with the MIXED procedure of SAS (2002–2012; SAS Institute, Inc. Cary, NC, USA). Analyses were first undertaken using a randomized complete block design with a 4 x 2 factorial for the four levels of carnosine and the two levels of H_2_O_2_. The presence of interaction forced the partitioned analysis of the effect of carnosine for each level of peroxide. Therefore, to determine the effect of carnosine in the absence of H_2_O_2_-induced oxidative stress (basal condition), a one-way ANOVA was carried out, followed by a Student’s t-test using a Dunnett correction for comparing the 10, 25 and 50 mM carnosine treatments (10, 50, 100 and 150 mM for the xCELLigence experiments) with the 0 mM carnosine control. For cells that were exposed to H_2_O_2_, a one-way ANOVA was performed, followed by a Student’s t-test using a Dunnett correction for comparing the 10, 25 and 50 mM carnosine treatments with the 0 mM carnosine + 0.3 mM H_2_O_2_ treatment. A comparison (specific contrast) between the 0 mM carnosine + 0 mM H_2_O_2_ control (Ctr.) and the 0 mM carnosine + 0.3 mM H_2_O_2_ treatments was also conducted to asses the specific effect of H_2_O_2_ in the absence of carnosine. Statistical analyses were performed and followed by the usual verification of the normality of the residuals (Shapiro-Wilk tests) to validate the results of the ANOVA. Statistical significance was set at *P*≤0.05 and tendencies at 0.05<*P*≤0.10.

## Results and discussion

### Carnosine affect the viability and proliferation of myoblasts subjected or not to H_2_O_2_-induced oxidative stress

The xCELLigence system, which allow real-time measurements of cell impedance (Fig 1A) was used to determine the carnosine concentrations to be used in our myoblast primary cell culture model. When compared with the 0 mM carnosine treatment, the rate of myoblast proliferative growth, as determined by the slope between two given time-points, was lower with the addition of 100 mM (*P*≤0.05) and 150 mM (*P*≤0.001) carnosine for 48 h (Fig 1B) and lower with 50 mM (*P*≤0.01), 100 mM (*P*≤0.001) and 150 mM (*P*≤0.001) carnosine over a 92 h growing period (Fig 1C). These results clearly demonstrate the cytotoxic effect of carnosine with supra-physiologic concentrations (e.g. 50 (92 h), 100 and 150 mM). Based on these results, it was decided to use 0, 10, 25 and 50 mM carnosine (for 48 h) in all subsequent assays. The 10 and 25 mM carnosine concentrations are within the observed physiological range for pig skeletal muscles [1], whereas the 50 mM carnosine represents a supra-physiologic concentration. In comparison, the observed baseline carnosine content in human skeletal muscle was 7.25 ± 1.47 mM in the soleus and 10.16 ± 1.91 mM in the gastrocnemius muscles when using proton magnetic resonance spectroscopy analyses [10].

**Fig 2:**
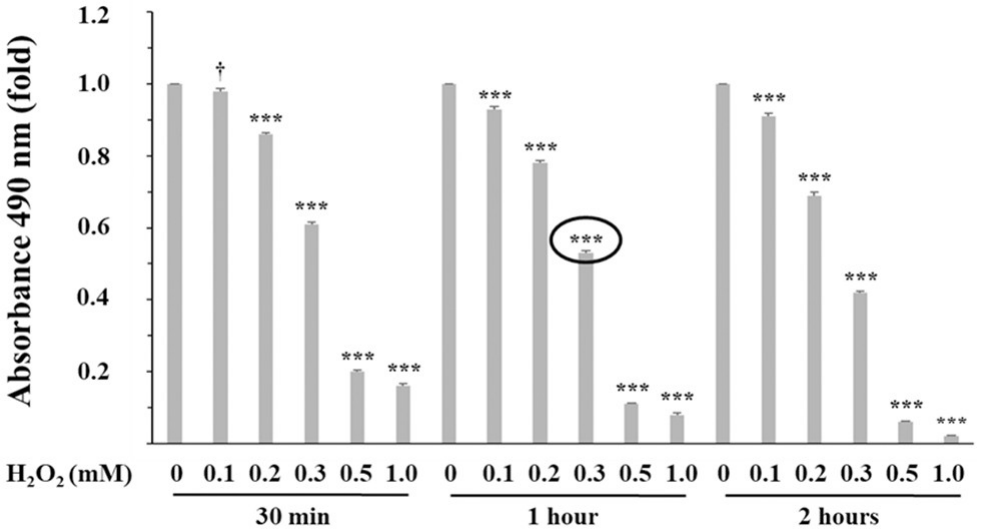
Effect of hydrogen peroxide (H_2_O_2_) on porcine myoblast viability. After 48 h in growth medium, cultured myoblasts were treated with H_2_O_2_ (0, 0.1, 0.2, 0.3, 0.5 and 1.0 mM) for 30 min, 1 h or 2 h and viability was assessed using the CellTiter 96^®^ AQueous One Solution Cell Proliferation Assay (MTS assay). Each H_2_O_2_ treatment was compared to the 0 mM H_2_O_2_ control, which was given a value of 1. The 0.3 mM H_2_O_2_ and 1 h condition (circle) was selected for all H_2_O_2_-induced oxidative stress analyses. Results are presented as mean absorbance values of triplicate from four independent experiments. SEM at 30 min = 0.01, 1 h = 0.01, 2 h = 0.01. ^†^0.05<*P*<0.1; ***P≤0.001.

The effect of H_2_O_2_ concentrations on cell viability was monitored using the MTS assay as a surrogate assay (Fig 2). These results show a dose-dependent viability decrease with increasing H_2_O_2_ concentrations and, this is true for all investigated time periods (30 min, 1 h and 2 h). The 0.3 mM H_2_O_2_ and 1 h condition was selected for this study since it results in approximately 50% cell mortality, which leaves enough room to observe the beneficial effects of carnosine pre-treatment.

**Fig 3:**
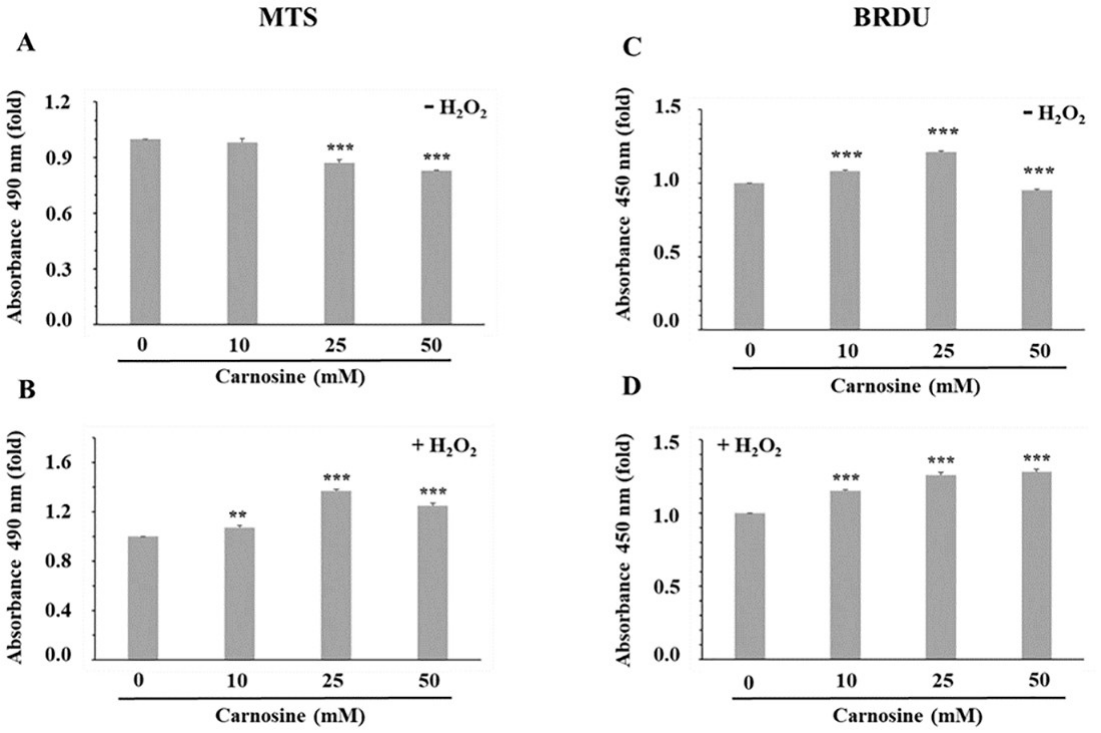
Effect of carnosine on porcine myoblast viability (A, B) and proliferation (C, D). (A, C) Effect of carnosine treatment (0, 10, 25 and 50 mM, 48 h) without H_2_O_2_ (-H_2_O_2_). The mean values of each carnosine concentration were compared to the 0 mM carnosine treatment using a Dunnett test. (B, D) Effect of carnosine pre-treatment (0, 10, 25 and 50 mM, 48 h) before H_2_O_2_-induced oxidative stress (0.3 mM, 1 h; + H_2_O_2_). The effect of carnosine in the presence of an H_2_O_2_-induced oxidative stress was determined by comparing each dose of carnosine to the 0 mM carnosine+ H_2_O_2_ treatment (Dunnett test). MTS = 3-(4,5-dimethyl-thiazol-2-yl)-5-(3-carboxymethoxyphenyl)-2-(4-sulfophenyl)-2H-tetrazolium (MTS) assay; BRDU = Bromodeoxyuridine (BRDU) assay. Values correspond to means ± SEM of n = 4 independent experiments in triplicates. MTS SEM without H_2_O_2_ = 0.02 (A) and with H_2_O_2_ = 0.01 (B). BRDU SEM without H_2_O_2_ = 0.02 (C) and with H_2_O_2_ = 0.01 (D). **P≤0.01; ***P≤0.001.

The effect of carnosine treatments, followed or not by an H_2_O_2_-induced oxidative stress, on porcine myoblast viability was then investigated. The addition of 25 and 50 mM carnosine for 48 h resulted in a significant decrease of myoblasts viability (Fig 3A; 25 and 50 mM *vs*. 0 mM, *P*≤0.001). On the opposite, pre-treating cells with 10, 25 and 50 mM carnosine for 48 h before inducing an oxidative stress increased myoblasts viability when compared with the 0 mM carnosine + H_2_O_2_ treatment (Fig 3B; *P*≤0.01). A similar protective effect of carnosine has been reported in rat primary chondrocyte cultures, where carnosine concentrations as low as 10 μM were able to reduce cell death induced by H_2_O_2_ [25]. Our results demonstrate that carnosine may have a dual effect on myoblasts viability, with a cytoprotective effect in cells subjected to high oxidative stress (Fig 3B) and a decrease of myoblast viability when exposed to high carnosine concentrations (Fig 3A).

In this study, myoblasts proliferation increased with 10 mM and 25 mM carnosine when compared with the 0 mM treatment (Fig 3C; *P*≤0.001), whereas the 50 mM treatment decreased proliferation (*P*≤0.001). A similar cell proliferation pattern was reported by Vishnyakova et al. [26], with a gradual increase of human skin fibroblast proliferation with increasing carnosine concentrations (up to 15 mM) and a return to proliferation values similar to the 0 mM treatment at 30 mM carnosine. Previous studies have reported an increase of human myoblasts proliferation when 20 mM carnosine was added to the culture medium after 30 days of cell growth [17], whereas there was no effect of carnosine on sheep myoblasts proliferation [27]. However, in the latter study, the carnosine concentrations used were very low, which may account for the lack of effect. Increases in cell proliferation are also observed when myoblasts were pre-treated with 10, 25 and 50 mM carnosine for 48 h before inducing an oxidative stress (Fig 3D; *vs*. 0 mM carnosine + H_2_O_2_, *P*≤0.001), thus suggesting a protective effect of carnosine for myoblasts exposed to H_2_O_2_-induced oxidative stress. The reason why the 25 mM carnosine treatment (without H_2_O_2_) shows opposite results for the MTS (Fig 3A) and BRDU (Fig 3C) assays remains to be determined. The MTS assay measures the mitochondrial metabolic rate, which indirectly reflect cell viability. Because metabolic activities can be influenced by different treatment conditions, the MTS assay may therefore introduce some bias and may account for the observed discrepancies between cell viability and proliferation data.

### Carnosine reduces the accumulation of reactive oxygen species and oxidative damage in myoblasts subjected or not to H_2_O_2_-induced oxidative stress

**Fig 4:**
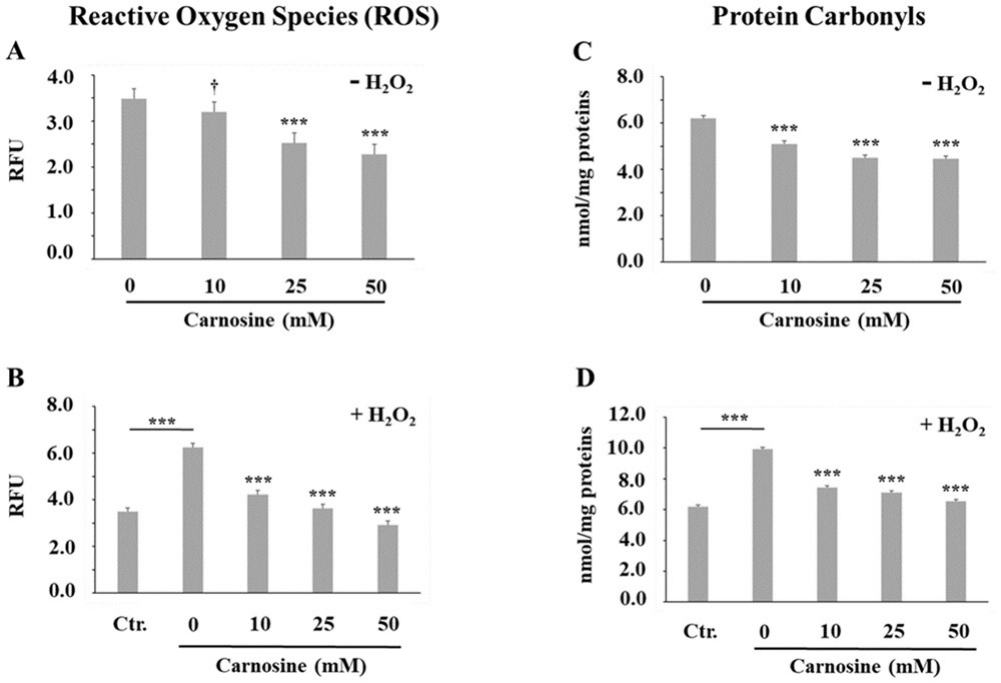
Effect of carnosine on the accumulation of intracellular reactive oxygen species (ROS) and protein carbonyls in porcine myoblasts. (A, C) Effect of carnosine treatment (0, 10, 25 and 50 mM, 48 h) without H_2_O_2_ (-H_2_O_2_). The mean values of each carnosine concentration were compared to the carnosine 0 mM treatment using a Dunnett test. (B, D) Effect of carnosine pre-treatment (0, 10, 25 and 50 mM, 48 h) before H_2_O_2_-induced oxidative stress (0.3 mM, 1 h; + H_2_O_2_). Ctr. = no carnosine and no H_2_O_2_. The effect of H_2_O_2_ on ROS and protein carbonyls accumulation was determined by specific contrast analyses between the Ctr. and 0 mM carnosine + H_2_O_2_ treatments (horizontal bars). The effect of carnosine in the presence of an H_2_O_2_-induced oxidative stress was determined by comparing each dose of carnosine to the carnosine 0 mM + H_2_O_2_ treatment (Dunnett test). Values correspond to means ± SEM of n = 4 independent experiments in duplicate. ROS SEM without H_2_O_2_ = 0.22 (A) and with H_2_O_2_ = 0.17 (B). Protein carbonyls SEM without H_2_O_2_ = 0.12 (C) and with H_2_O_2_ = 0.11 (D). ^†^0.05<*P*<0.1; ***P≤0.001.

Carnosine’s ability to scavenge ROS and to reduce the cellular content of protein carbonyls has been previously reported [1] and, in this study, we provide the first evidence of a similar protective effect of carnosine in porcine myoblast cells. Indeed, in basal conditions the addition of 10 mM (0.05<*P*≤0.1), 25 mM (*P*≤0.001) and 50 mM (*P*≤0.001) carnosine reduced the accumulation of intracellular ROS (Fig 4A) and protein carbonyls (Fig 4C; *P*≤0.001) when compared with the 0 mM carnosine control. As expected, the addition of H_2_O_2_ to the cell culture media increased the ROS and protein carbonyls content (Fig 4B and 4D; H_2_O_2_ + 0 mM carnosine *vs*. Ctr., *P*≤0.001) and pre-treating cells with 10, 25 and 50 mM carnosine prevented these increases (*vs*. H_2_O_2_ + 0 mM carnosine, *P*<0.001). Carnosine also prevented H_2_O_2_-induced increases of protein carbonyls in rat skeletal muscle homogenates [28] as well as H_2_O_2_-induced increases of ROS in INS-1E and MIN6 pancreatic beta cell lines [29]. Using a cellular model of diabetic glucolipotoxicity, Cripps et al. [30] also demonstrated that carnosine is an effective scavenger of reactive oxygen species in INS -1 cells and C2C12 myotubes. The ability of carnosine to directly scavenge reactive carbonyl species, such as the 4-hydroxynonenal, and reduce damage to proteins has been demonstrated *in vitro* using ovalbumin, α-crystalin and ubiquitin target protein models [31, 32]. Ma et al. [14] observed a reduction of protein carbonyls in the *longissimus* muscle of growing pigs supplemented with 100 mg carnosine/kg of diet for 8 weeks when compared with control pigs. However, since muscle carnosine content was not quantified in that paper [14], it remains unclear whether the observed effect is due to an increase of muscle carnosine.

### Addition of carnosine to myoblast cultures prevents the H_2_O_2_-associated decline of SOD and GPX activities

**Fig 5:**
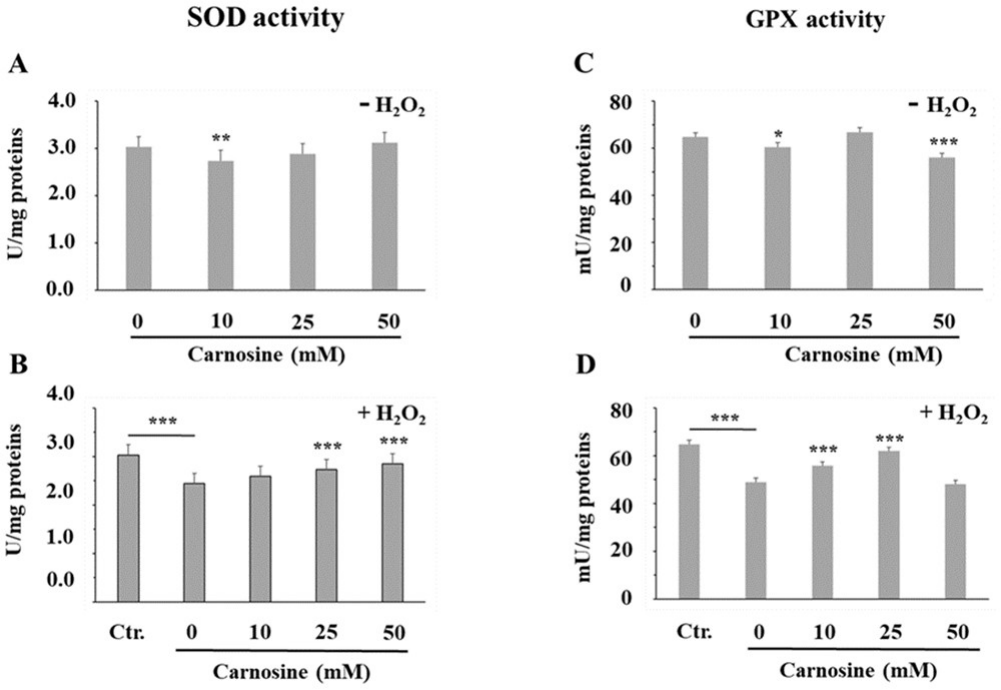
Effect of carnosine on intracellular superoxide dismutase (SOD, A and B) and glutathione peroxidase (GPX, C and D) activities in porcine myoblast cells. (A and C) Effect of carnosine treatment (0, 10, 25 and 50 mM, 48 h) without H_2_O_2_-induced oxidative stress (-H_2_O_2_). The means of each carnosine concentration were compared to the carnosine 0 mM treatment using a Dunnett test. (B and D) Effect of carnosine pre-treatment (0, 10, 25 and 50 mM, 48 h) before H_2_O_2_-induced oxidative stress (0.3 mM, 1 h; + H_2_O_2_). Ctr. = no carnosine and no H_2_O_2_. The effect of H_2_O_2_ on SOD and GPX activities was determined by specific contrast analyses between the Ctr. and 0 mM carnosine + H_2_O_2_ treatments (horizontal bars). The effect of carnosine in the presence of an H_2_O_2_-induced oxidative stress was determined by comparing each dose to the carnosine 0 mM + H_2_O_2_ treatment (Dunnett test). Values correspond to means ± SEM of n = 4 independent experiments in duplicate. SOD activity SEM without H_2_O_2_ = 0.22 (A) and with H_2_O_2_ = 0.21 (B). GPX activity SEM without H_2_O_2_ = 1.90 (C) and with H_2_O_2_ = 1.71 (D). *P≤0.05; **P≤0.01; ***P≤0.001.

Different detoxification mechanisms exist to prevent cellular oxidative damage. Among these, SOD and GPX are key antioxidant enzymes that suppress or reduce the formation of reactive oxygen species in order to maintain the cellular redox homeostasis [33]. Dietary supplementation with carnosine was shown to increase muscle SOD activity in pigs and muscle SOD and GPX activities in broilers [14, 34]. Conversely, carnosine supplementation had no effect on GPX activity or the antioxidant status of rats [35]. It remains unclear whether carnosine has a direct or indirect effect on antioxidant enzymes, but the inverse relationship recently found between the carnosine + anserine content and the SOD activity in chicken and hummingbird muscles rather suggest an indirect effect [36]. In the present study, addition of 10 mM carnosine to the cell culture media decreased the SOD activity (*vs*. 0 mM carnosine, *P*<0.01), whereas the 25 and 50 mM treatments had no effect (Fig 5A). At physiological concentrations, carnosine presents SOD-like activity, being able to form charge transfer complexes with superoxide anions (O_2_·^-^) [37]. Copper-carnosine complexes also demonstrated SOD-like activity in human blood neutrophils treated with phorbol miresitate acetate (PMA) [38]. Therefore, carnosine action may result in lower requirements for cellular SOD activity, which may explain the observed reduction in SOD activity with carnosine (Fig 5A). However, this was only observed with the 10 mM carnosine treatment (9.5% reduction), thus suggesting a limited effect of carnosine on SOD activity in the absence of H_2_O_2_-induced oxidative stress.

A 19.5% reduction in total SOD activity was observed with addition of H_2_O_2_ alone (Fig 5B; 0 mM carnosine *+* H_2_O_2_ *vs*. Ctr; *P*<0.001) and pre-treating cells with 25 mM and 50 mM carnosine prevented this H_2_O_2_-mediated activity reduction (Fig 5B; *vs*. 0 mM carnosine, *P*<0.01). This reduction in myoblast SOD activity with H_2_O_2_ may be caused by a protein fragmentation effect, as previously observed when incubating purified recombinant human Cu,Zn-SOD with H_2_O_2_ [39]. In accordance with our results, these authors also reported a protective effect of carnosine on H_2_O_2_-mediated Cu,Zn-SOD fragmentation, possibly through the hydroxyl radical (•OH) scavenging activity of carnosine [39].

In this study, glutathione peroxidase activity was also affected by carnosine. In basal conditions, there was a decrease in total GPX activity when myoblasts were treated with 10 mM (*P*<0.05) and 50 mM (*P*<0.001) carnosine (Fig 5C; *vs*. 0 mM carnosine). It was earlier demonstrated that carnosine is able to reduce lipid hydroperoxides into their corresponding alcohol [40], an activity that is also performed by glutathione peroxidases. The observed decrease in cellular GPX activity in the absence of H_2_O_2_ may be due, in part, to the effect of carnosine in reducing the cellular oxidative stress, as observed with the reduction of ROS and protein carbonyls biomarkers.

The observed decrease in GPX activity (24.6%) when myoblasts were exposed to H_2_O_2_-induced oxidative stress was expected (Fig 5D; 0 mM carnosine *+* H_2_O_2_ *vs*. Ctr; *P*<0.001) since this has been reported in various cell systems [41, 42]. Pre-treating cells with carnosine at physiological concentrations prevented this H_2_O_2_-induced GPX activity reduction (Fig 5D; 10 mM and 25 mM carnosine *vs*. 0 mM carnosine, *P*<0.001), whereas no protective effect was observed with 50 mM carnosine. Additional work is needed to understand the reasons why supra-physiologic doses (50 mM) of carnosine have no protective effect on GPX activity, whereas physiological doses (10 and 25 mM) restore GPX activity in myoblasts exposed to H_2_O_2_-induced oxidative stress.

### Carnosine affects the mRNA abundance of genes involved in cellular defence against oxidative stress

To determine whether the protective effect of carnosine against oxidative stress might be regulated at the transcription level, the mRNA abundance of *NEF2L2*, a redox-activated transcription factor, was measured along with several antioxidant response element (ARE)-responsive genes [43]. In basal conditions (Table 2), the addition of carnosine increased the mRNA abundance of *NEF2L2* (50 mM, *P*<0.05), *PRDX3* (50 mM, *P*<0.05), *PRDX4* (25 and 50 mM, *P*<0.05) and *SOD2* (25 and 50 mM, *P*<0.01) when compared with the 0 mM carnosine treatment. Conversely, the addition of 10, 25 and 50 mM carnosine reduced the *GPX3* (vs. 0 mM carnosine, *P*<0.01) and *SOD3* (vs. 0 mM carnosine, *P*<0.001) gene expression, two isoforms located in the extracellular space where they are involved in the detoxification of H_2_O_2_ and O_2_·^-^, respectively [44, 45].

**Table 2:** Effect of carnosine on the relative mRNA abundance of antioxidant genes in porcine myoblast cells.

Genes^1^	Carnosine (mM)^2^				SEM	*P*-value
	0	10	25	50		
*CAT*	1.03	1.05	1.13	1.04	0.042	0.162
*GPX1*	1.02	1.01	1.04	1.09	0.032	0.252
*GPX3*	1.24	1.07*	1.04**	0.97**	0.077	0.002
*GPX4*	1.04	1.12	1.11	1.11	0.052	0.320
*GSR*	1.15	1.18	1.30	1.20	0.099	0.669
*NEF2L2*	0.99	1.10	1.20	1.37*	0.089	0.029
*PRDX1*	1.01	0.93	0.91	0.89	0.041	0.115
*PRDX3*	0.81	0.81	0.91	0.99*	0.062	0.014
*PRDX4*	0.87	0.96	1.09**	1.08*	0.047	0.009
*SOD1*	0.99	1.02	0.99	0.96	0.040	0.497
*SOD2*	0.92	0.99	1.08**	1.15**	0.044	<0.001
*SOD3*	1.36	0.70**	0.67**	0.77**	0.091	<0.001
*TXNRD1*	1.18	1.24	1.33	1.34	0.053	0.150
*TXNRD2*	1.37	1.30	1.39	1.28	0.068	0.261

^1^*CAT*, Catalase; *GPX1*, Glutathione Peroxidase 1; *GPX3*, Glutathione Peroxidase 3; *GPX4*, Glutathione Peroxidase 4; *GSR*, Glutathione-Disulfide Reductase; *NEF2L2*, Nuclear Factor, Erythroid 2 Like 2; *PRDX1*, Peroxiredoxin 1; *PRDX3*, Peroxiredoxin 3; *PRDX4*, Peroxiredoxin 4; *SOD1*, Superoxide Dismutase 1; *SOD2*, Superoxide Dismutase 2; *SOD3*, Superoxide Dismutase 3; *TXNRD1*, Thioredoxin Reductase 1; *TXNRD2*, Thioredoxin Reductase 2.

^2^Each value represents the mean of four experiments in triplicate.

*, *P*<0.05;

**, *P*<0.01;

***, *P*<0.001, when compared with the control treatment (0 mM carnosine and no H_2_O_2_).

When compared to the Ctr. treatment, the mRNA abundance of *NEF2L2* and several of its downstream antioxidant genes decreased when myoblasts were exposed to H_2_O_2_-induced oxidative stress (Table 3; *P*≤0.001 for *CAT*, *GPX1*, *GPX3*, *NEF2L2*, *PRDX3*, *SOD3*, *TXNRD1* and *TXNRD2*; *P*<0.05 for *GPX4* and *PRDX4*; tendencies for *GSR* and *SOD2*). On the other hand, the H_2_O_2_ treatment had no effect on *PRDX1* and *SOD1* mRNA levels (*P*>0.1). In accordance with these results, addition of 50 μM H_2_O_2_ to human umbilical vein endothelial cells (HUVEC) for 24 h decreased the expression levels of *CAT*, *GPX1*, *NFE2L2*, *TXRND1* and *TXRND2* and had no effect on *SOD1* and *SOD2* mRNA levels [46].

**Table 3:** Effect of carnosine on the relative mRNA abundance of antioxidant genes in porcine myoblasts subjected to H_2_O_2_-induced oxidative stress.

Genes^1^		Carnosine (mM)^3^				SEM	Carn^4^	H_2_O_2_^5^
	Ctr.^2^	0	10	25	50		*P*-value	*P*-value
*CAT*	1.10	0.78	0.82	1.06***	0.71	0.039	<0.001	<0.001
*GPX1*	1.10	0.85	0.99**	1.10***	0.90	0.050	<0.001	<0.001
*GPX3*	1.33	0.73	0.80	0.96***	0.69	0.067	<0.001	<0.001
*GPX4*	1.11	1.00	1.05	1.02	1.02	0.044	0.654	0.029
*GSR*	1.23	1.02	1.29*	1.18	1.18	0.086	0.113	0.077
*NEF2L2*	1.05	0.66	0.92*	0.90*	1.06***	0.084	0.003	<0.001
*PRDX1*	1.06	1.07	1.14	1.16*	0.97*	0.035	0.001	0.971
*PRDX3*	0.86	0.66	0.67	0.92***	0.81*	0.039	<0.001	0.001
*PRDX4*	0.93	0.79	0.87	0.99**	0.98**	0.043	0.003	0.017
*SOD1*	1.05	0.96	1.18**	1.07†	1.02	0.050	0.015	0.129
*SOD2*	0.98	0.89	0.93	0.97	0.91	0.060	0.405	0.091
*SOD3*	1.46	0.86	0.99	0.75	0.80	0.085	0.230	<0.001
*TXNRD1*	1.26	0.99	1.03	1.13†	0.94	0.081	0.097	0.001
*TXNRD2*	1.47	1.06	1.14†	1.02	0.96*	0.052	0.003	<0.001

^1^*CAT*, Catalase; *GPX1*, Glutathione Peroxidase 1; *GPX3*, Glutathione Peroxidase 3; *GPX4*, Glutathione Peroxidase 4; *GSR*, Glutathione-Disulfide Reductase; *NEF2L2*, Nuclear Factor, Erythroid 2 Like 2; *PRDX1*, Peroxiredoxin 1; *PRDX3*, Peroxiredoxin 3; *PRDX4*, Peroxiredoxin 4; *SOD1*, Superoxide Dismutase 1; *SOD2*, Superoxide Dismutase 2; *SOD3*, Superoxide Dismutase 3; *TXNRD1*, Thioredoxin Reductase 1; *TXNRD2*, Thioredoxin Reductase 2.

^2^Ctr., 0 mM carnosine and 0 mM H_2_O_2_.

^3^Each value represents the mean of four experiments in triplicate.

^†^, tendency;

*, *P*<0.05;

**, *P*<0.01;

***, *P*<0.001, when compared with the 0 mM carnosine + 0.3 mM H_2_O_2_ treatment (second column).

^4^Overall effect of carnosine in the presence of H_2_O_2_ (Carnosine 0, 10, 25 and 50 mM).

^5^Effect of the specific contrast (Carnosine 0 mM + 0.3 mM H_2_O_2_ vs. Ctr.).

In this study, we also observed increases in the mRNA abundance of *NEF2L2* and peroxidase enzymes (*CAT*, *GPX1*, *GPX3*, *PRDX1*, *PRDX3*, *PRDX4*) when myoblasts were treated with carnosine before inducing the oxidative stress (Table 3; *vs*. 0 mM carnosine + H_2_O_2_, *P*<0.01). These results suggest that the observed beneficial effect of carnosine on intracellular ROS detoxification (Fig 4B) may be mediated through an upregulation of *NEF2L2* mRNA abundance and some of its downstream ARE containing antioxidant genes. Using a rat model of CCL4-induced hepatic injury, Alsheblak et al. [47] reported a similar cytoprotective effect of carnosine through the upregulation of hepatic Nrf2 level, which is encoded by the *NEF2L2* gene. Carnosine also increased *NEF2L2* mRNA levels in mouse podocytes (MPC5) cell cultures exposed to high glucose oxidative stress [48]. Of interest, the *GPX1* gene expression profile is similar to the GPX activity profile (Fig 5D), thus suggesting that the carnosine protective effect on GPX activity may be related with the observed increase in *GPX1* transcripts level. However, this does not seem to be the case for the SOD activity (Fig 5B) since carnosine had no effect on *SOD2* and *SOD3* gene expression (Carn *P*-values >0.1) and a limited effect on *SOD1* expression (Carn *P*<0.05) (Table 3).

### Carnosine affects the p38 MAPK, p44/42 MAPK and mTOR intracellular signaling pathways in myoblasts

Elevated intracellular ROS content is known to activate mitogen-activated protein kinases (MAPKs) that will in turn trigger different signal transduction cascades. Of these MAPKs, the best characterized ones are the p38, ERK1/2 (p44/42 MAPK) and JNK serine/threonine kinases that regulate cell survival and cell death [49]. Kefalogianni and Gaitanaki [50] previously demonstrated that exogenous H_2_O_2_ activates the p38, ERK1/2 (p44/42 MAPK) and JNK signaling pathways in murine C2 myoblasts and, carnosine was found to prevent the activation of ERK1/2 in rats neurons subjected to H_2_O_2_-induced oxidative stress [51]. Carnosine was also able to reduce the activation of p38 and ERK1/2 MAPKs in rat mesangial cells exposed to high glucose oxidative stress [52].

**Fig 6:**
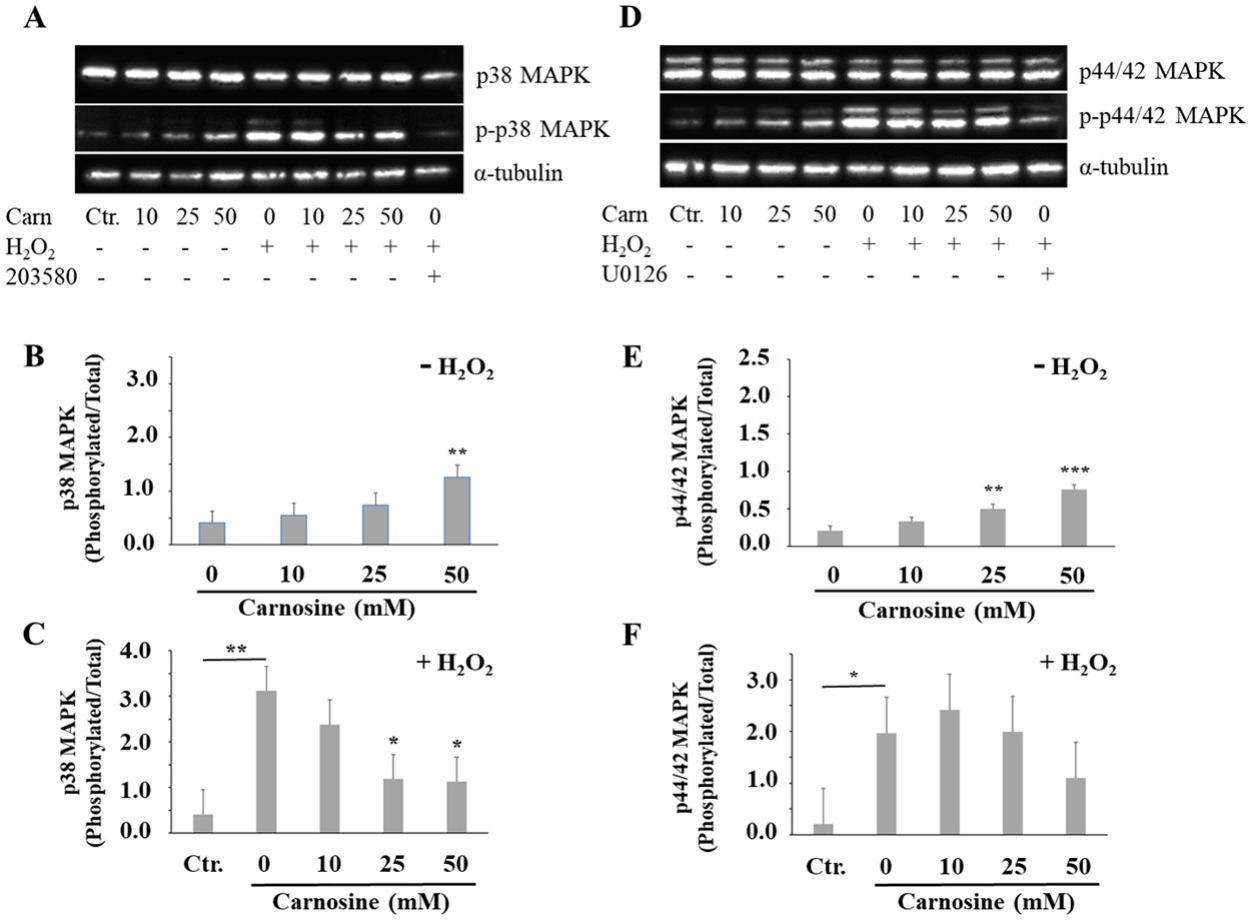
Effect of carnosine on the activation of p38 (A, B, C) and p44/42 (D, E, F) MAPK-dependent pathways in porcine myoblasts. Representative immunoblots showing total and phosphorylated p38 MAPK (A) and p44/42 MAPK (D). Values were first normalized with corresponding α-Tubulin data. These results were then used to present data as a ratio of phosphorylated protein/total protein. (B, E) Effect of carnosine treatment (0, 10, 25 and 50 mM, 48 h) without H_2_O_2_-induced oxidative stress (-H_2_O_2_). (C, F) Effect of carnosine pre-treatment (0, 10, 25 and 50 mM, 48 h) before H_2_O_2_-induced oxidative stress (0.3 mM, 1 h; + H_2_O_2_), the effect of H_2_O_2_ on p38 MAPK and p44/42 MAPK phosphorylation was determined by specific contrast analyses between the Ctr. and 0 mM carnosine + H_2_O_2_ treatments (horizontal bars). Ctr. = no carnosine and no H_2_O_2_. Values correspond to means ± SEM of n = 4 independent experiments. p38 MAPK SEM without H_2_O_2_ = 0.22 (B) and with H_2_O_2_ = 0.54 (C). p44/42 MAPK SEM without H_2_O_2_ = 0.06 (E) and with H_2_O_2_ = 0.69 (F). 203580 and U0126 are specific inhibitors of p38 MAPK and p44/42 MAPK phosphorylation, respectively. *P≤0.05; **P≤0.01; *** *P*≤0.001.

In the absence of H_2_O_2_-induced oxidative stress (Fig 6A, 6B, 6D and 6E), the highest carnosine concentrations increased the phosphorylation of p38 MAPK (50 mM vs. 0 mM carnosine, *P*<0.01) and p44/42 MAPK (25 and 50 mM vs. 0 mM carnosine, *P*<0.01). In contrast, there was no effect of carnosine on the activation of SAPK/JNK (*P*>0.1; S1 Fig). The p38 MAPK is usually described as being more responsive to stress stimuli, whereas ERK1/2 is more associated with growth factors activation and is required for MM14 mouse myoblasts proliferation [53]. However, in the presence of sustained activation, ERK1/2 can also provoke cell death [54]. This may be the case with the carnosine treatments since myoblasts exposed to 50 mM carnosine for 92 h had reduced proliferation rates (vs. 0 mM carnosine; *P*<0.01), as measured with the xCELLigence system, whereas there was no significant effect at 48 h (Fig 1).

Moreover, treating cells with 50 mM carnosine for 48 h increased p38 and p44/42 MAPK phosphorylation, with concomitant reductions in cell viability and proliferation (Fig 3A and 3C; Fig 6B and 6E).

As expected, there were significant increases in p38 MAPK (Fig 6A and 6C; *P*<0.01), p44/42 MAPK (Fig 6D and 6F; *P*<05) and SAPK/JNK (*P*<0.05; S1 Fig) phosphorylation in H_2_O_2_-treated myoblasts (vs. Ctr. treatment). Pre-treatment with 25 mM or 50 mM carnosine before inducing the oxidative stress attenuated the p38 phosphorylation when compared with the 0 mM carnosine + H_2_O_2_ treatment (Fig 6A and 6C; *P*<0.05). However, the carnosine pre-treatment did not prevent the activation of p44/42 MAPK (Fig 6D and 6F; *P*>0.1) and SAPK/JNK (*P*>0.1; S1 Fig) phosphorylation in myoblasts exposed to H_2_O_2_. Collectively, our results suggest that the observed protective effects of carnosine in H_2_O_2_-treated myoblasts are mediated, at least in part, through the p38 MAPK signaling pathway.

**Fig 7:**
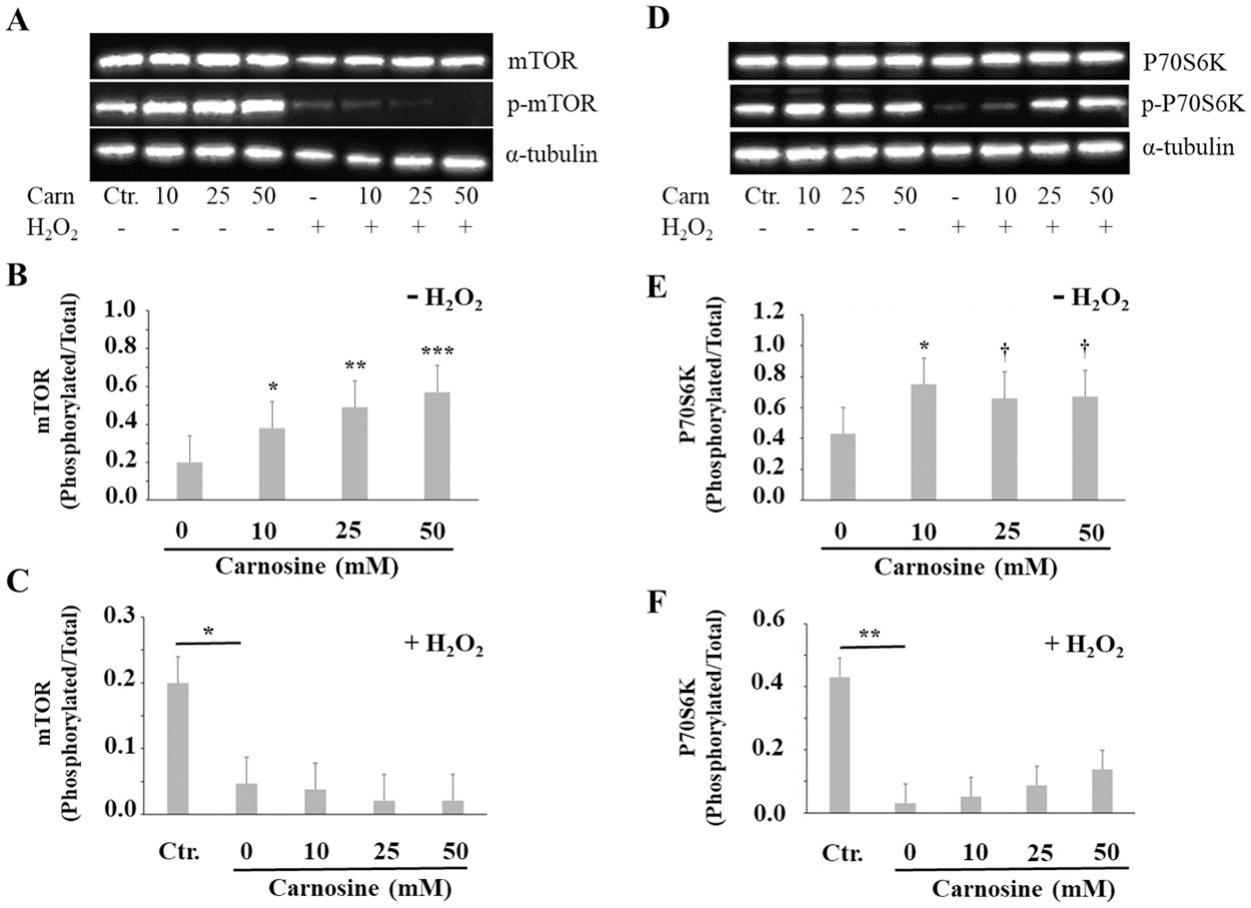
Effect of carnosine on the activation of mTOR (A, B, C) and its downstream effector p70S6K (D, E, F) in porcine myoblasts. Representative immunoblots showing total and phosphorylated mTOR (A) and p70S6K (D). Values were first normalized with corresponding α-Tubulin data. These results were then used to present data as a ratio of phosphorylated protein/total protein. (B, E) Effect of carnosine treatment (0, 10, 25 and 50 mM, 48 h) without H_2_O_2_-induced oxidative stress (-H_2_O_2_). (C, F) The effect of H_2_O_2_ on mTOR and p70S6K phosphorylation was determined by specific contrast analyses between the Ctr. and 0 mM carnosine + H_2_O_2_ treatments (horizontal bars). The effect of carnosine pre-treatment (0, 10, 25 and 50 mM, 48 h) before H_2_O_2_-induced oxidative stress (0.3 mM, 1 h; + H_2_O_2_) was determined by comparing each dose to the carnosine 0 mM + H_2_O_2_ treatment (Dunnett test). Ctr. = no carnosine and no H_2_O_2_. Values correspond to means ± SEM of n = 4 independent experiments. mTOR SEM without H_2_O_2_ = 0.14 (B) and with H_2_O_2_ = 0.04 (C). p70S6K SEM without H_2_O_2_ = 0.17 (E) and with H_2_O_2_ = 0.06 (F). ^†^0.05<*P*<0.1; *P≤0.05; **P≤ 0.01; ***P≤ 0.001.

The mammalian target of rapamycin (mTOR) is a serine/threonine kinase that senses different cellular and extracellular signals to control proliferation, growth and survival [55]. Growth factors signaling lead to the activation of mTOR, which will in turn phosphorylates P70S6K and 4E-BP1, two downstream substrates known to regulate protein translation initiation and progression [56], whereas energy or nutrient depletion and stress signals suppress mTOR signaling. Supra-physiological doses of carnosine were found to inactivate the mTOR signaling pathway in human gastric carcinoma cells [57], but had no effect on mTOR phosphorylation in human glioblastoma cells [58] and in rat brain homogenates [59]. In this study, there was a clear dose-response increase in mTOR phosphorylation with the addition of 10 mM (*P*<0.05), 25 mM (*P*<0.01) and 50 mM (*P*<0.001) carnosine, when compared with the 0 mM treatment (Fig 7A and 7B). Carnosine also increased the phosphorylation of P70S6K (Fig 7D and 7E), with a significant effect at 10 mM (vs. 0 mM, *P*<0.05) and tendencies at 25 mM and 50 mM carnosine.

An increase in 4E-BP1 phosphorylation was also observed when myoblasts were treated with 25 mM carnosine (vs. 0 mM, tendency *P* = 0.081; S1 Fig). In addition to the well known effect of branched amino acids on the activation of the mTORC1 complex, it was recently demonstrated that accumulation of histidine also increase mTOR (Ser2481) phosphorylation in bovine mammary epithelial cells [60]. Moreover, the inactivation of SLC15A4, a carnosine and histidine transporter, decreased the mTOR (Ser2481) and p70S6K (Thr389) phosphorylation in B-cells [61]. Therefore, it remains to be determined whether the observed effects of carnosine on mTOR and its downstream substrates, p70S6K and 4E-BP1, in porcine myoblasts are due to carnosine itself or to histidine, once carnosine is cleaved into L-histidine and β-alanine by carnosinases. An indirect effect of carnosine through the activation of ERK1/2 (p44/42) MAPK is also possible since crosstalk between mTOR and the RAS-ERK1/2 signaling pathways has been reported before [62] and, similar increases in p-mTOR and p-p44/42 MAPK were observed in myoblasts exposed to increasing concentrations of carnosine (Figs 6E and 7B).

Significant decreases in mTOR (*P*<0.05), P70S6K (*P*<0.01) and 4E-BP1 (*P*<0.001; S1 Fig) phosphorylation were observed in myoblasts subjected to H_2_O_2_-induced oxidative stress (Fig 7A, 7C, 7D and 7F; Ctr. vs. 0 mM carnosine + H_2_O_2_). Similar observations were made in neuronal cells where dose-response decreases of mTOR, P70S6K and 4E-BP1 phosphorylation were found with increasing H_2_O_2_ concentrations [63]. Carnosine pre-treatment of myoblasts did not prevent the H_2_O_2_-mediated decreases of mTOR, P70S6K and 4E-BP1 phosphorylation (Fig 7A, 7C, 7D and 7F) suggesting a lack of effect of carnosine on mTOR signaling pathway when cells are exposed to oxidative stress.

### Carnosine affects myoblasts intracellular oxygen consumption rate

**Fig 8:**
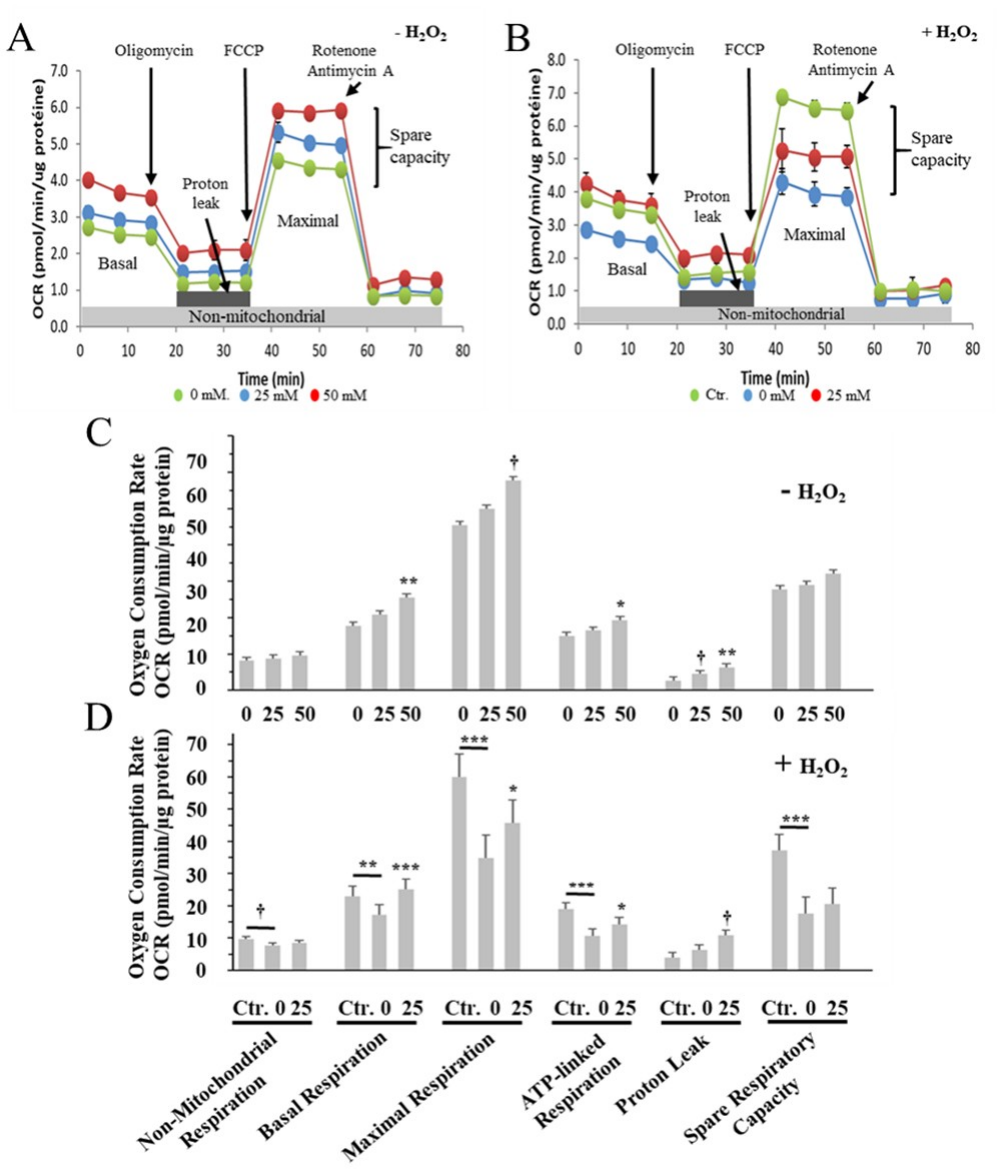
Effect of carnosine on cellular oxygen consumption rate (OCR) in porcine myoblasts. (A, C) Effect of carnosine treatment (0, 25 and 50 mM, 48 h) without H_2_O_2_-induced oxidative stress (-H_2_O_2_). The mean values of each carnosine concentration were compared to the carnosine 0 mM treatment using a Dunnett test. (B, D) Effect of carnosine pre-treatment (0 *vs*. 25 mM, 48 h) before H_2_O_2_-induced oxidative stress (0.3 mM, 1 h; + H_2_O_2_). Ctr. = no carnosine and no H_2_O_2_. The effect of H_2_O_2_ on OCR parameters was also determined by specific contrast analyses between the Ctr. and 0 mM carnosine + H_2_O_2_ treatments (horizontal bars). Values correspond to means ± SEM of n = 5 independent experiments performed in duplicate. OCR SEM without H_2_O_2_ (C): Non-mitochondrial respiration = 0.56, Basal respiration = 1.52, Maximal respiration = 5.16, ATP-linked respiration = 1.57, Proton leak = 0.84, Spare respiratory capacity = 3.9; OCR SEM with H_2_O_2_ (D): Non-mitochondrial respiration = 0.71, Basal respiration = 3.17, Maximal respiration = 7.11, ATP-linked respiration = 2.13, Proton leak = 1.61, Spare respiratory capacity = 5.02. ^†^0.05<*P*<0.1; *P≤0.05; **P≤0.01; ***P≤0.001.

Increase in cellular oxidative stress is known to affect mitochondrial functions, including disruption of mitochondrial membrane potential and ATP production [64]. Since carnosine was found to reduce intracellular ROS accumulation (Fig 4A and 4B), we hypothesized that the addition of carnosine to myoblasts might affect the mitochondrial bioenergetics properties. We therefore conducted analyses to assess different cellular and mitochondrial oxygen consumption rate parameters (Fig 8). In basal conditions (Fig 8A and 8C), the addition of 50 mM carnosine significantly increased the basal respiration (*vs* 0 mM carnosine; *P*<0.01), which is the sum of the ATP-linked, proton leak and non-mitochondrial OCR. Injection of oligomycin, an ATP synthase inhibitor, then revealed an increase of mitochondrial ATP-linked (*P*<0.05) and proton leak (*P*<0.01) OCR in cells exposed to 50 mM carnosine (*vs* 0 carnosine). Under conditions where basal respiration is affected by treatment, as observed in the current study, it is important to consider the apparent proton leak, which is the percentage of proton leak relative to its corresponding basal respiration. In this study, the apparent proton leak increased with the addition of 25 mM (21.63%; *P*<0.01) and 50 mM (24.56%; *P*<0.001) carnosine when compared with the 0 mM control treatment at 15.44%. In comparison, previous studies have reported similar proton leak values of 20% and 22%, relative to the basal respiration, for untreated C_2_C_12_ mouse myoblasts [65, 66]. An increase in apparent proton leak can be associated with mitochondrial inner membrane and/or electron transport chain (ETC) damages, and it could also be a sign of increased uncoupling protein (UCP) activity or electron slippage [67]. However, the observed increase in maximal respiration OCR after injection of FCCP, for myoblasts treated with 50 mM carnosine (*vs* 0 mM carnosine; 0.05<*P*<0.1), does not support the fact that carnosine may cause harmful damages to the mitochondrial ETC.

This is based on previous literature reporting that an increase in maximal respiration OCR indicates an increased cellular capacity to respond to additional energy demand [67]. Therefore, this increase in maximal respiration with 50 mM carnosine may rather suggest that carnosine in preserving mitochondrial integrity.

Myoblasts exposed to H_2_O_2_-induced oxidative stress had reduced basal, maximal and ATP-linked respiration (Fig 8B and 8D; 0 mM carnosine *vs* Ctr.; *P*<0.01). These results demonstrate that the addition of H_2_O_2_ to myoblasts induced mitochondrial dysfunction. Myoblasts treated with H_2_O_2_ also had higher apparent proton leak when compared with the Ctr. treatment (36.49% vs 16.42%; *P*<0.01). Accumulation of excessive cellular ROS, as reported in the present study with H_2_O_2_ (Fig 4B), is known to induce mitochondrial injuries and promote proton leak [64]. Such increase in proton leak can then activate feedback mechanisms leading to reduction of mitochondrial ROS production in order to limit further damage to mitochondria [68, 69]. Therefore, it will be of interest to investigate whether the observed increase in proton leak with the addition of H_2_O_2_ may be associated with the activation of a similar protective feedback loop.

Cells treated with 25 mM carnosine before H_2_O_2_ treatment had higher basal (*P*<0.001), maximal (*P*<0.05) and ATP-liked (*P*<0.05) respiration when compared with the 0 mM carnosine + H_2_O_2_ treatment (Fig 8B and 8D). These results demonstrate that carnosine is able to reduce damages caused by H_2_O_2_, thus suggesting a protective role towards mitochondrial bioenergetics. However, it remains to be determined whether this is a direct role, such as protection of mitochondrial inner membrane damages and increased proton leak to reduce mitochondria ROS production, or an indirect role through the reduction of cellular ROS. The higher spare respiratory capacity observed in control (Ctr.) when compared with H_2_O_2_ treated cells (with or without carnosine) suggests that H_2_O_2_ has a detrimental effect on the ability of porcine myoblasts to cope with large increases in ATP turnover and that carnosine did not improve this stress response. In this study, similar apparent proton leak values were observed between the 0 mM (36.49%) and 25 mM (42.67%) carnosine treatments (*P*>0.1) in myoblasts subjected to H_2_O_2_-induced oxidative stress. This would suggest that the observed positive effect of carnosine on basal, maximal and ATP-linked respiration (Fig 8D) is not associated with an increase of mitochondrial proton leak. In accordance with results from this study, the protective effect of carnosine on mitochondrial functions was recently reported. For example, carnosine dietary supplementation preserved brain mitochondrial ATP content and decreased mitochondrial depolarization in Mn-exposed mice [70] and, reduced mitochondrial depolarization in the sperm of Pb-exposed rats [71]. Using cultured rat astrocytes under ischemic conditions, Shen et al. [72] also reported a protective effect of carnosine on mitochondrial bioenergetics. On the other hand, carnosine was found to have the opposite effect in cancer cells, with reduction of cellular ATP production in malignant glioma [73] and colon cancer cells [74]. Decreased basal OCR and ATP-linked OCR were also observed in HeLa cells [75] and gastric cancer cells [76] with a 20 mM carnosine treatment. Collectively, these results suggest a dual role for carnosine on mitochondrial functions, having a protective effect in non transformed cells such as the myoblasts and, decreasing mitochondrial bioenergetics in cancer cells. One possible explanation for this discrepancy may be associated with the ability of carnosine to suppress glycolysis and with the fact that tumor cells are more dependent on glycolysis for ATP production when compared with non transformed cells [77].

### Concluding remarks

Results from the present study clearly demonstrate that carnosine acts differently in myoblasts exposed to an oxidative stress challenge and in basal conditions. The only exception is for the ROS and protein carbonyls data where carnosine decreased the accumulation of reactive oxygen species and reduced oxidative damage in both basal and oxidative stress conditions. Our results also suggest that the protective effect of carnosine in oxidative stress conditions is mediated at different levels. At the transcription level, carnosine increases the *NEF2L2* mRNA abundance and its downstream ARE responsive peroxidase genes *CAT*, *GPX1*, *GPX3*, *PRDX1*, *PRDX3* and *PRDX4*. Our results also suggest a protective role of carnosine towards mitochondrial bioenergetics that may be mediated through the observed reduction in cellular ROS. However, further work is needed to better characterize this latter mechanism.

In this study, we also demonstrated that carnosine modulates different intracellular signaling pathways in myoblasts under basal and oxidative stress conditions. Indeed, carnosine prevents the H_2_O_2_-mediated activation of p38 MAPK, possibly through a mechanism involving the reduction of cellular ROS, whereas it activates the p38 MAPK in myoblasts under basal conditions. The dose-response activation of mTOR by carnosine suggests an effect on myoblasts proliferation, growth and survival under basal conditions. However, it remains to be determined whether this activation is mediated through a direct effect of carnosine or an indirect effect due to a crosstalk between the ERK1/2 (p44/42) MAPK and mTOR signaling pathways.

Although carnosine is able to reduce oxidative stress biomarkers, activate the intracellular mTOR pathway and increase cellular oxygen consumption rate under basal conditions, current cell viability and proliferation data also suggest that supra-physiologic carnosine concentrations are likely cytotoxic for myoblasts. On the other hand, the protective effect of carnosine in myoblasts subjected to H_2_O_2_-induced oxidative stress was observable at all tested concentrations, with no harmful effect of supra-physiologic concentrations on cell viability or proliferation. These observations support the fact that carnosine acts differently in myoblasts under basal and oxidative stress conditions.

## Supporting information

S1 Fig Effect of carnosine on the activation of 4E-BP1 (A, B, C) and SAPK/JNK (D, E, F) in porcine myoblasts. Representative immunoblots showing total and phosphorylated 4E-BP1 (A) and SAPK/JNK (D). Values were first normalized with corresponding α-Tubulin data. These results were then used to present data as a ratio of phosphorylated protein/total protein. (B, E) Effect of carnosine treatment (0, 10, 25 and 50 mM, 48 h) without H_2_O_2_-induced oxidative stress (-H_2_O_2_). (C, F) The effect of H_2_O_2_ on 4E-BP1 and SAPK/JNK phosphorylation was determined by specific contrast analyses between the Ctr. and 0 mM carnosine + H_2_O_2_ treatments (horizontal bars). The effect of carnosine pre-treatment (0, 10, 25 and 50 mM, 48 h) before H_2_O_2_-induced oxidative stress (0.3 mM, 1 h; + H_2_O_2_) was determined by comparing each dose to the carnosine 0 mM + H_2_O_2_ treatment (Dunnett test). Ctr. = no carnosine and no H_2_O_2_. Values correspond to means ± SEM of n = 4 independent experiments. 4E-BP1 SEM without H_2_O_2_ = 0.48 (B) and with H_2_O_2_ = 0.17 (C). SAPK/JNK SEM without H_2_O_2_ = 0.02 (E) and with H_2_O_2_ = 0.62 (F). ^†^0.05<*P*<0.1; *P≤0.05; **P≤ 0.01; ***P≤ 0.001.
(TIF)

S1 Raw images (PDF)
