# Supplementary figures and images for: Characterisation of intracellular molecular mechanisms modulated by carnosine in porcine myoblasts under basal and oxidative stress conditions
Source: PLoS One. 2020 Sep 18;15(9):e0239496. doi: 10.1371/journal.pone.0239496 (PMC7500635; doi:10.1371/journal.pone.0239496)

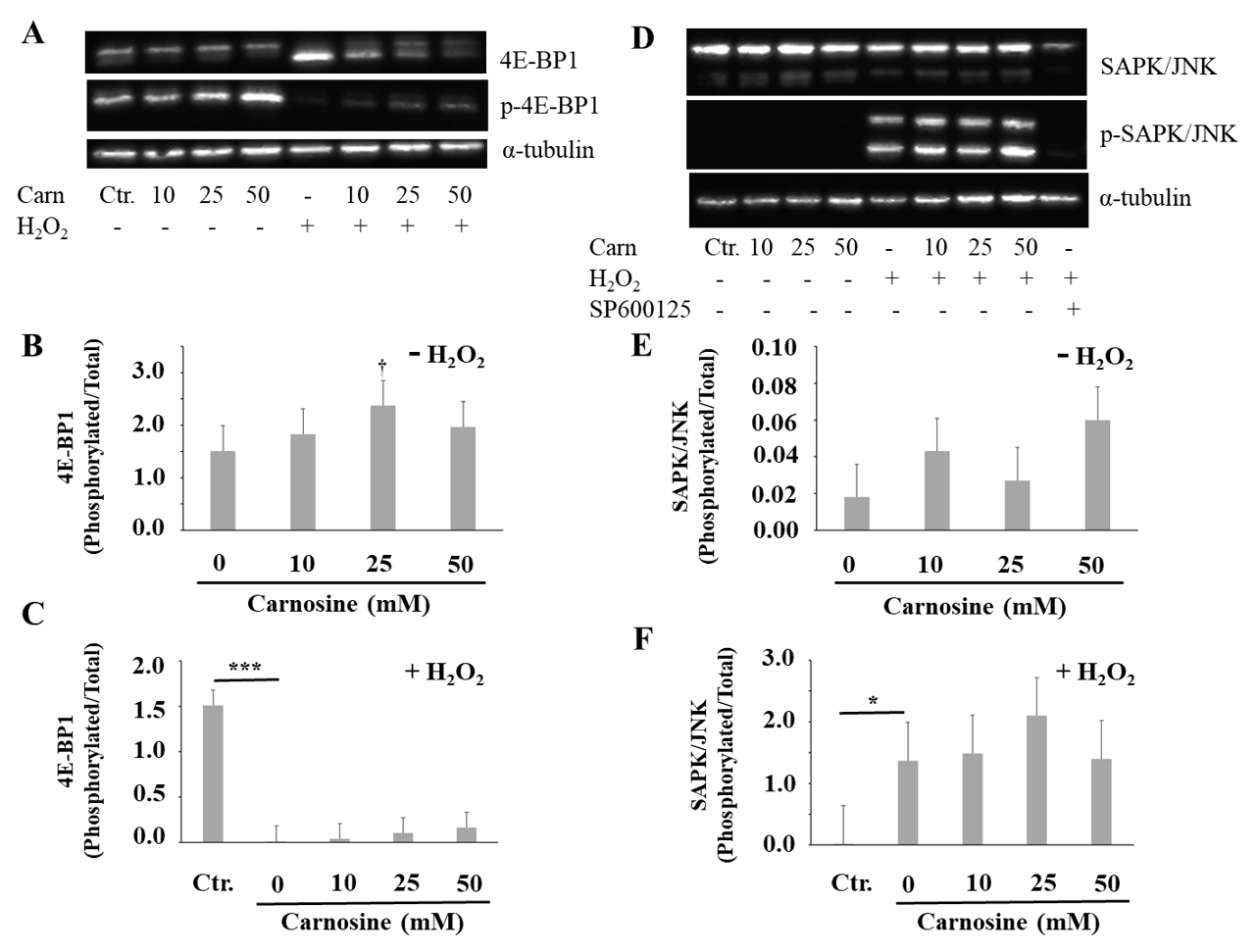

Supplement: S1 Fig — Representative immunoblots showing total and phosphorylated 4E-BP1 (A) and SAPK/JNK (D). Values were first normalized with corresponding α-Tubulin data. These results were then used to present data as a ratio of phosphorylated protein/total protein. (B, E) Effect of carnosine treatment (0, 10, 25 and 50 mM, 48 h) without H2O2-induced oxidative stress (-H2O2). (C, F) The effect of H2O2 on 4E-BP1 and SAPK/JNK phosphorylation was determined by specific contrast analyses between the Ctr. and 0 mM carnosine + H2O2 treatments (horizontal bars). The effect of carnosine pre-treatment (0, 10, 25 and 50 mM, 48 h) before H2O2-induced oxidative stress (0.3 mM, 1 h; + H2O2) was determined by comparing each dose to the carnosine 0 mM + H2O2 treatment (Dunnett test). Ctr. = no carnosine and no H2O2. Values correspond to means ± SEM of n = 4 independent experiments. 4E-BP1 SEM without H2O2 = 0.48 (B) and with H2O2 = 0.17 (C). SAPK/JNK SEM without H2O2 = 0.02 (E) and with H2O2 = 0.62 (F). †0.05<P<0.1; *P≤0.05; **P≤ 0.01; ***P≤ 0.001. (TIF) [file pone.0239496.s001.tif]
