# Supplementary material for: Characterisation of intracellular molecular mechanisms modulated by carnosine in porcine myoblasts under basal and oxidative stress conditions
Source: PLoS One. 2020 Sep 18;15(9):e0239496. doi: 10.1371/journal.pone.0239496 (PMC7500635; doi:10.1371/journal.pone.0239496)
Supplement: S1 Raw images — (PDF) [file pone.0239496.s002.pdf]

Western Blot raw image

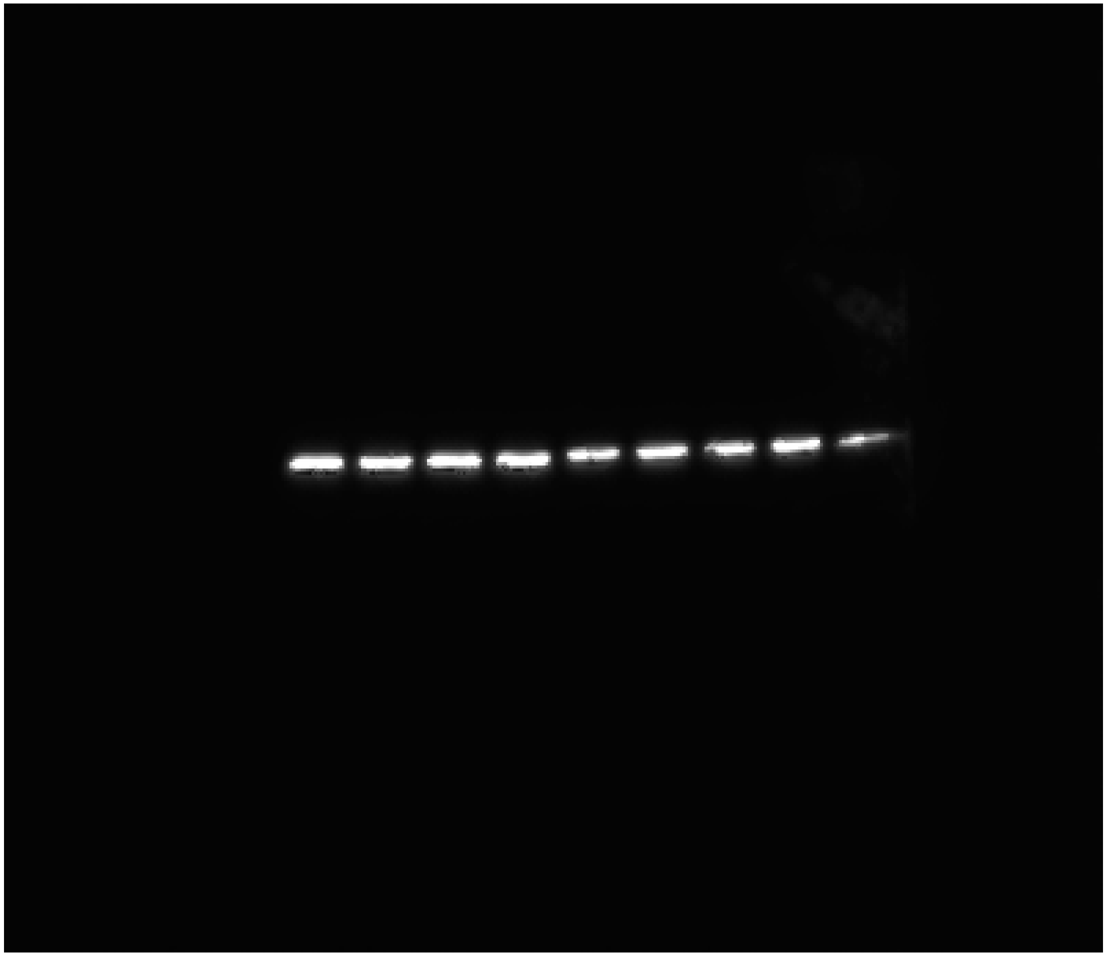

Fig. 6A
p38 MAPK

Western Blot raw image

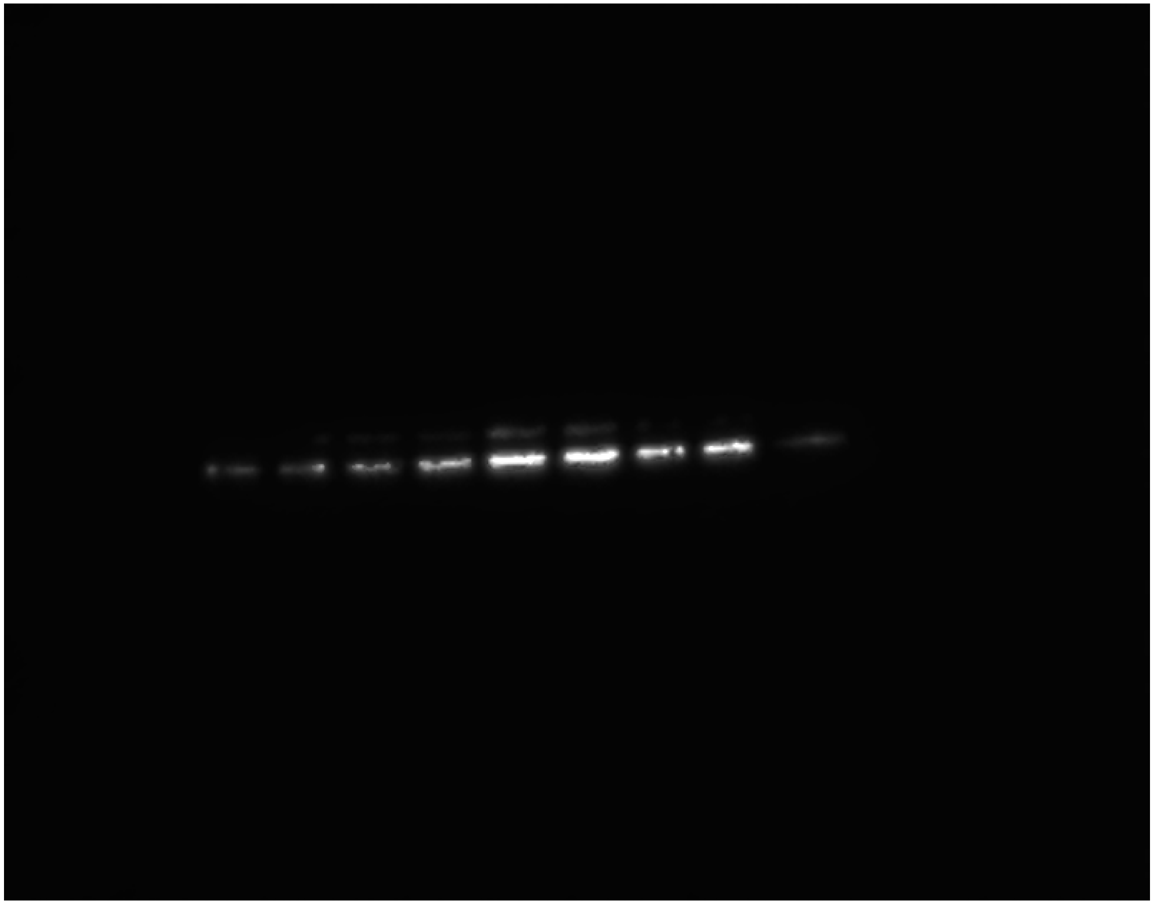

Fig. 6A
P-p38 MAPK

Western Blot raw image

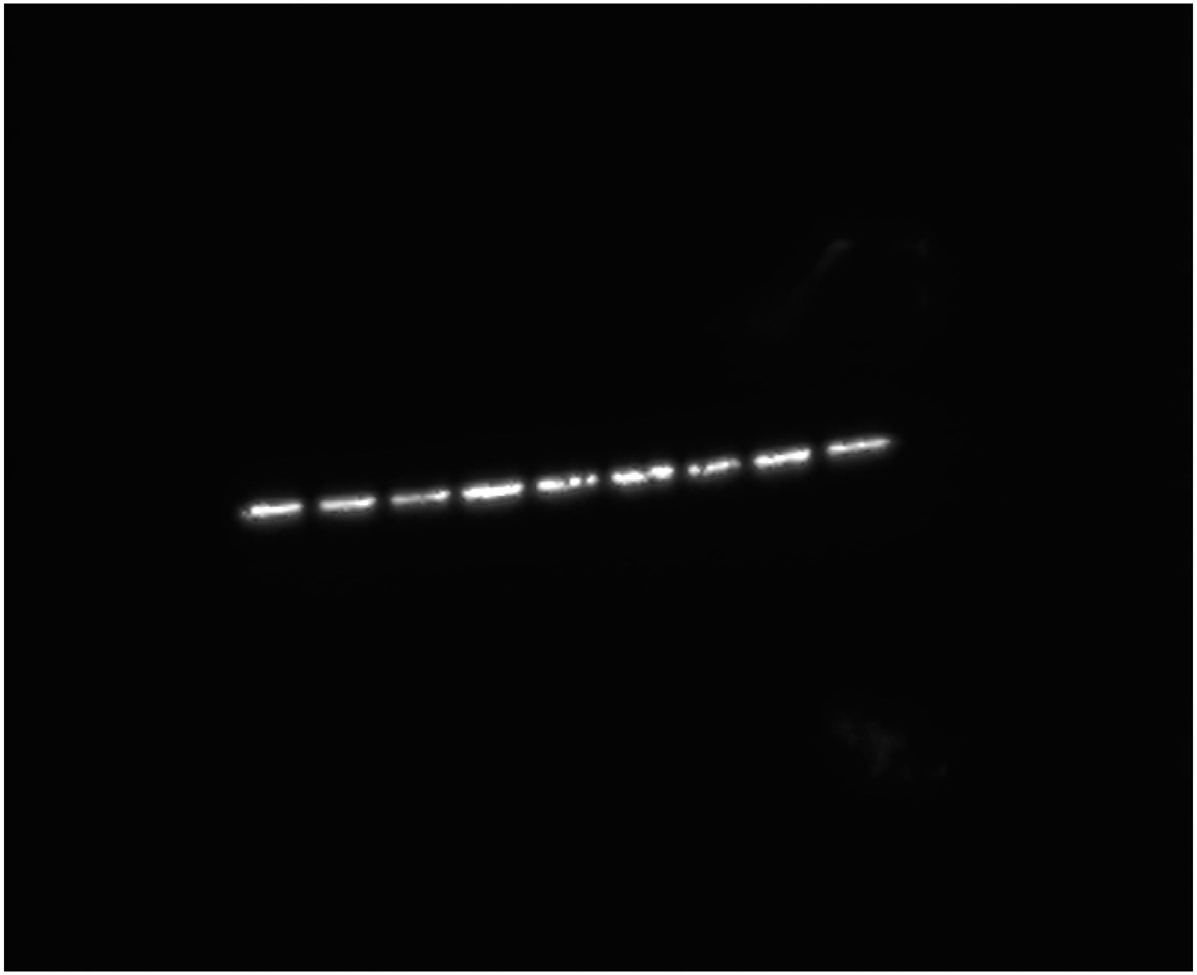

Fig. 6A
α-tubulin

Western Blot raw image

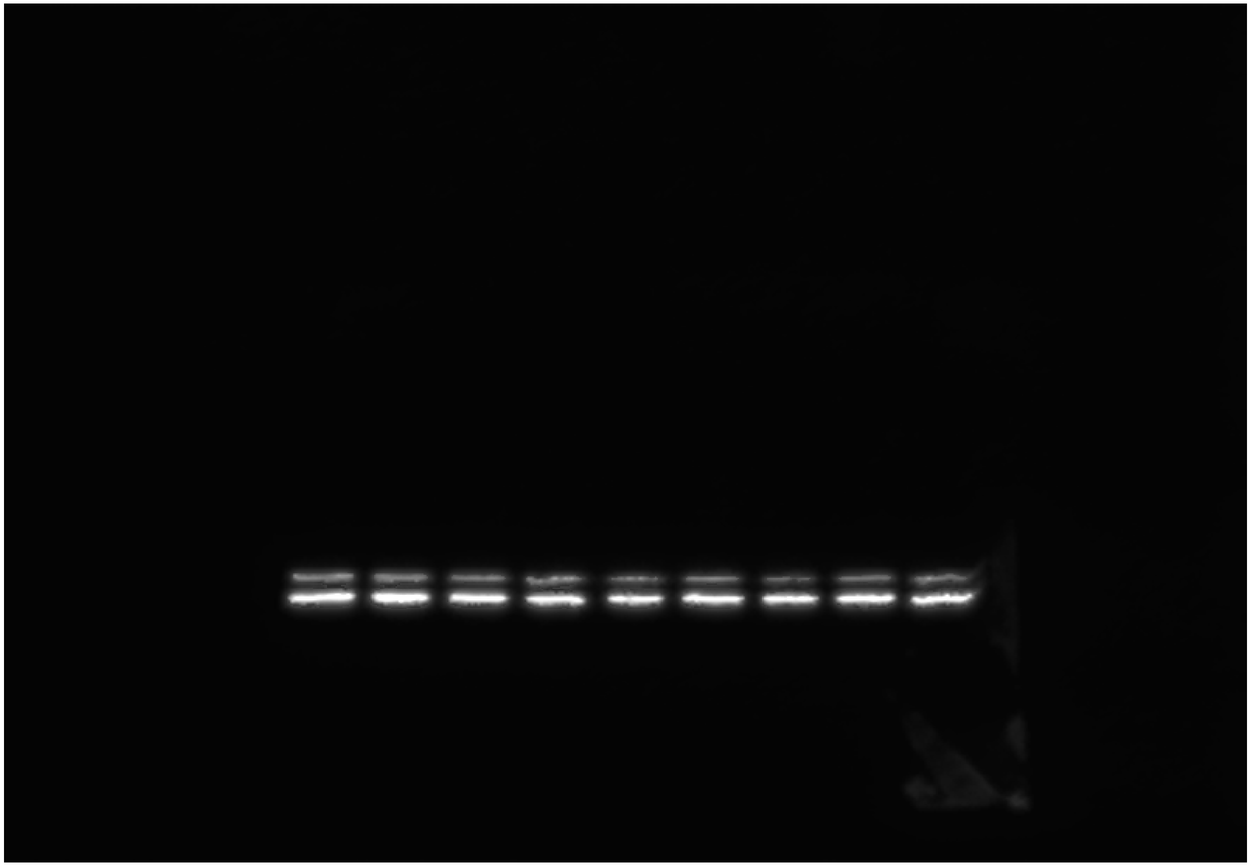

Fig. 6D
p44/42 MAPK

Western Blot raw image

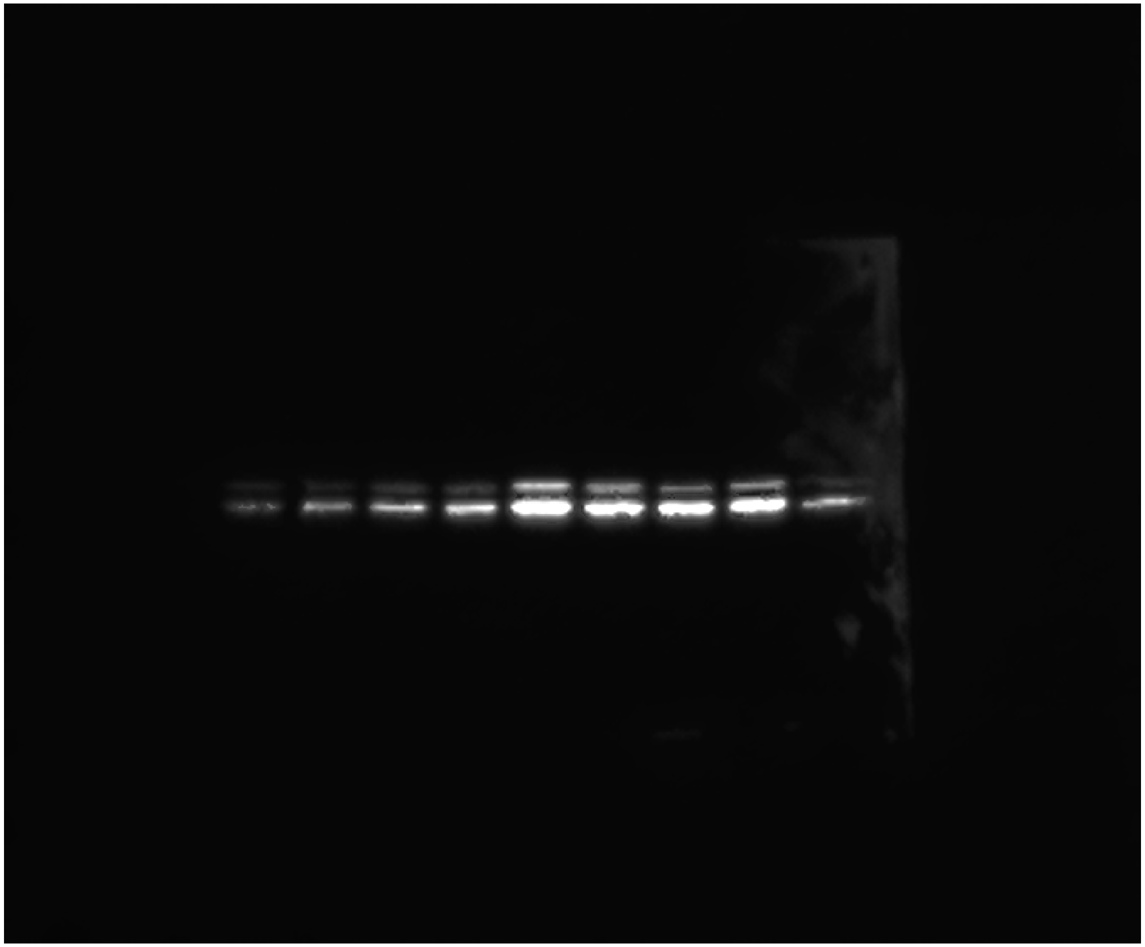

Fig. 6D
p-p44/42 MAPK

Western Blot raw image

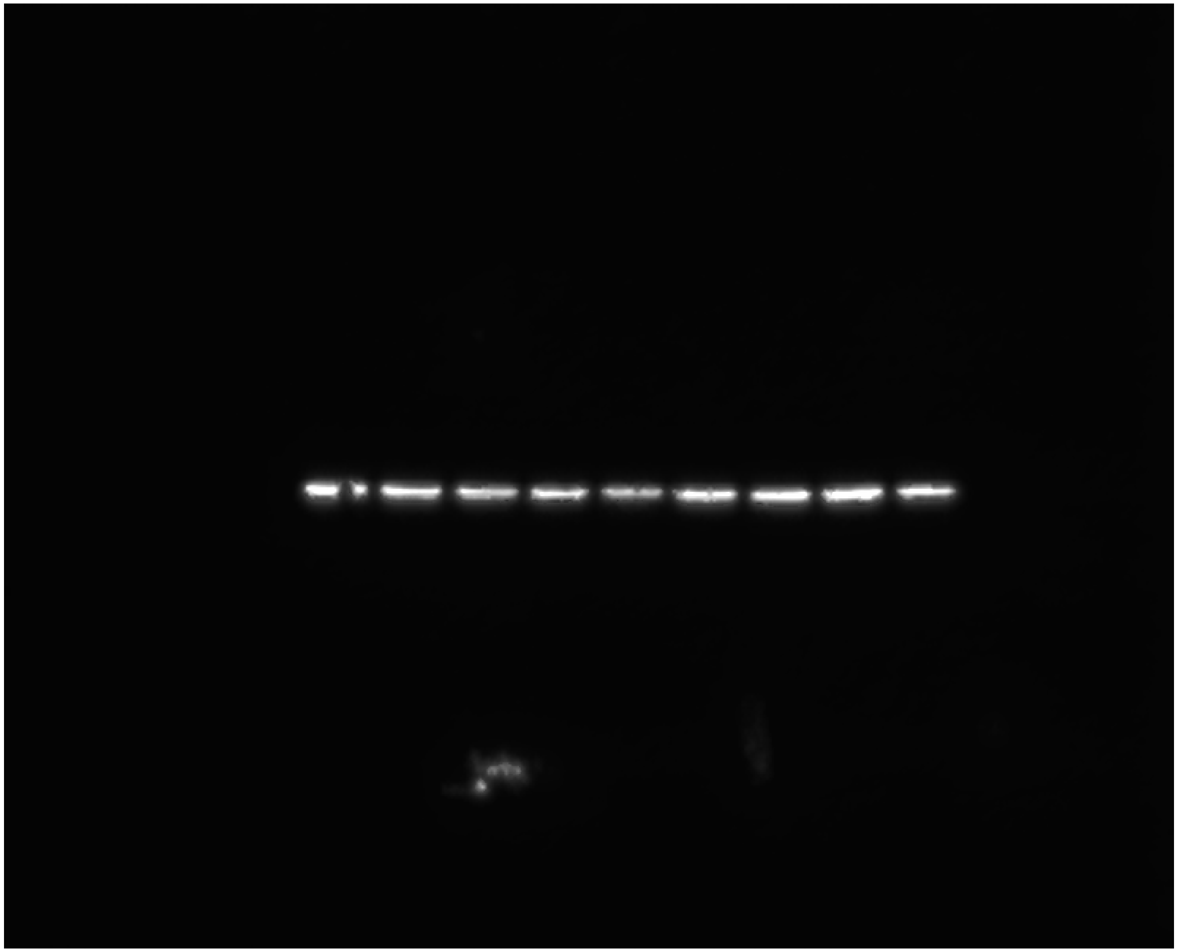

Fig. 6D
α-tubulin

Western Blot raw image

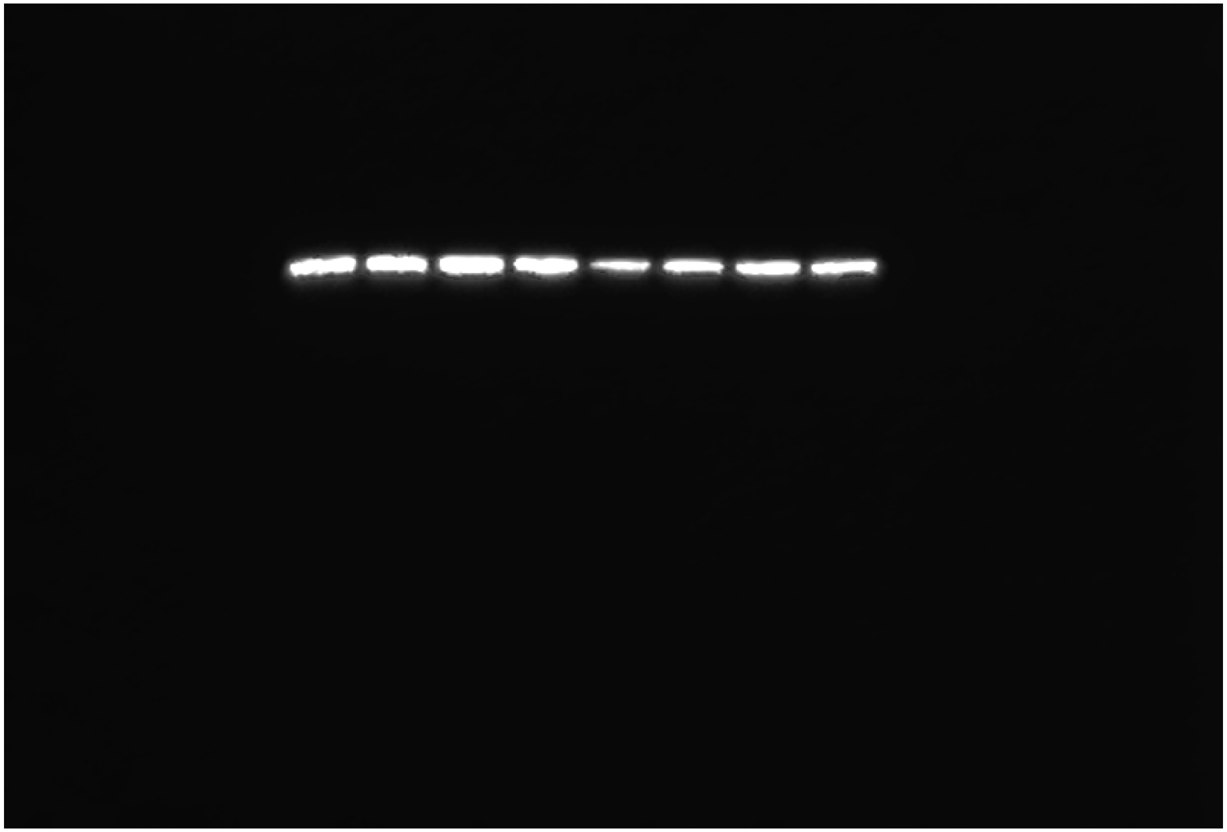

Fig. 7A
mTOR

Western Blot raw image

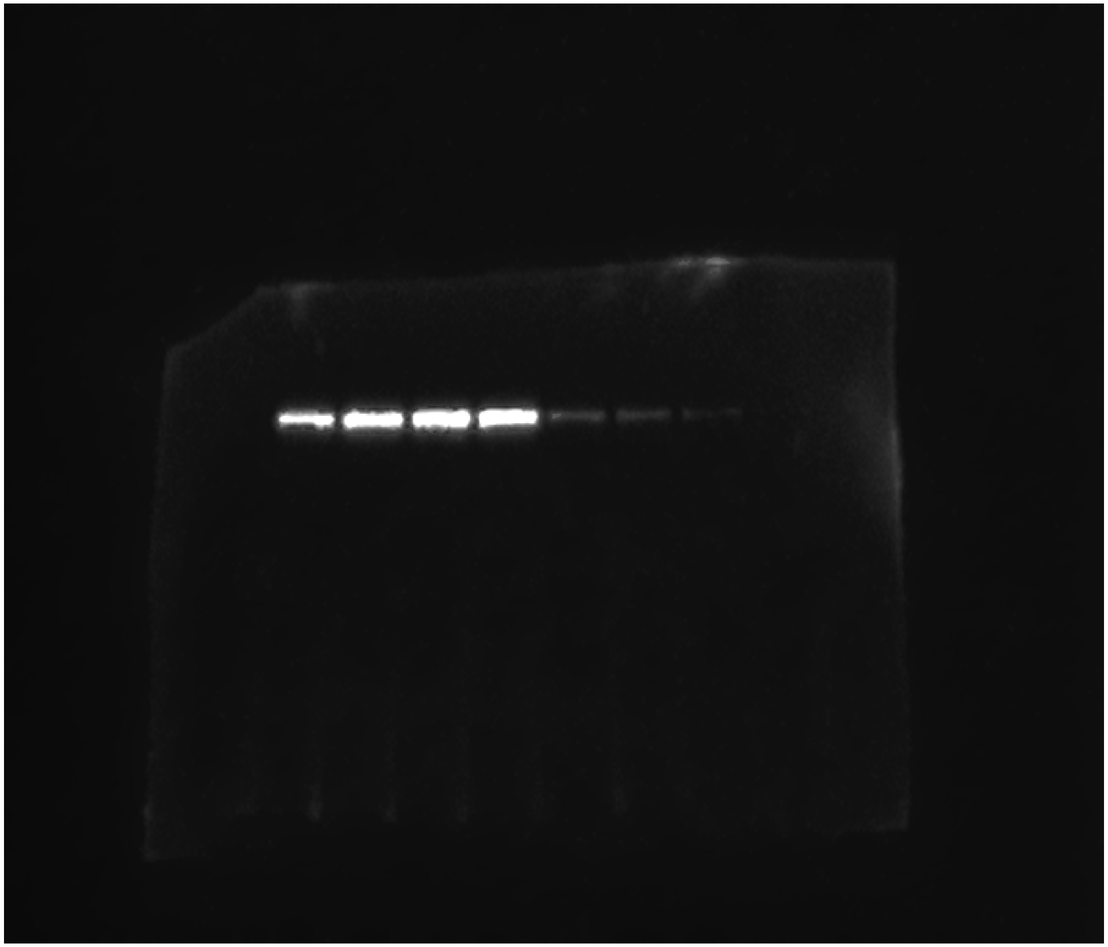

Fig. 7A
p-mTOR

Western Blot raw image

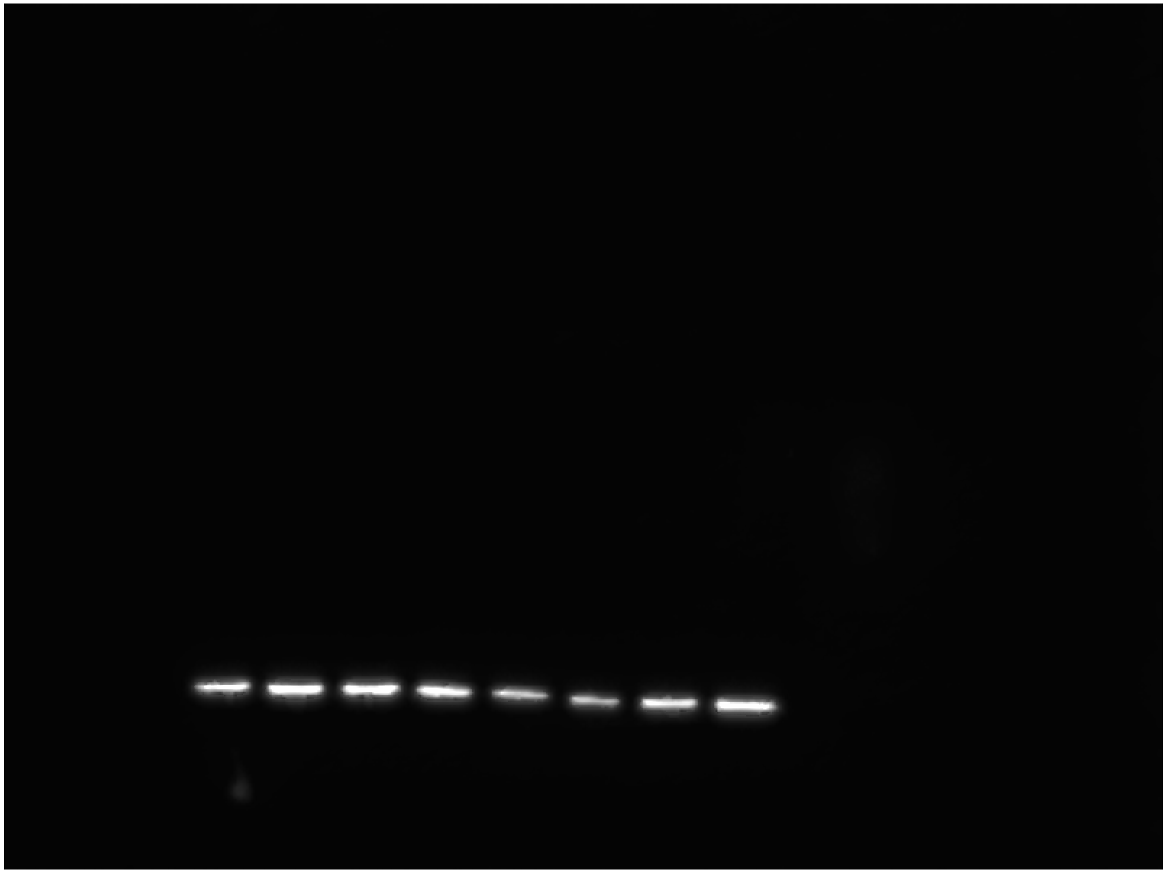

Fig. 7A
α-tubulin

Western Blot raw image

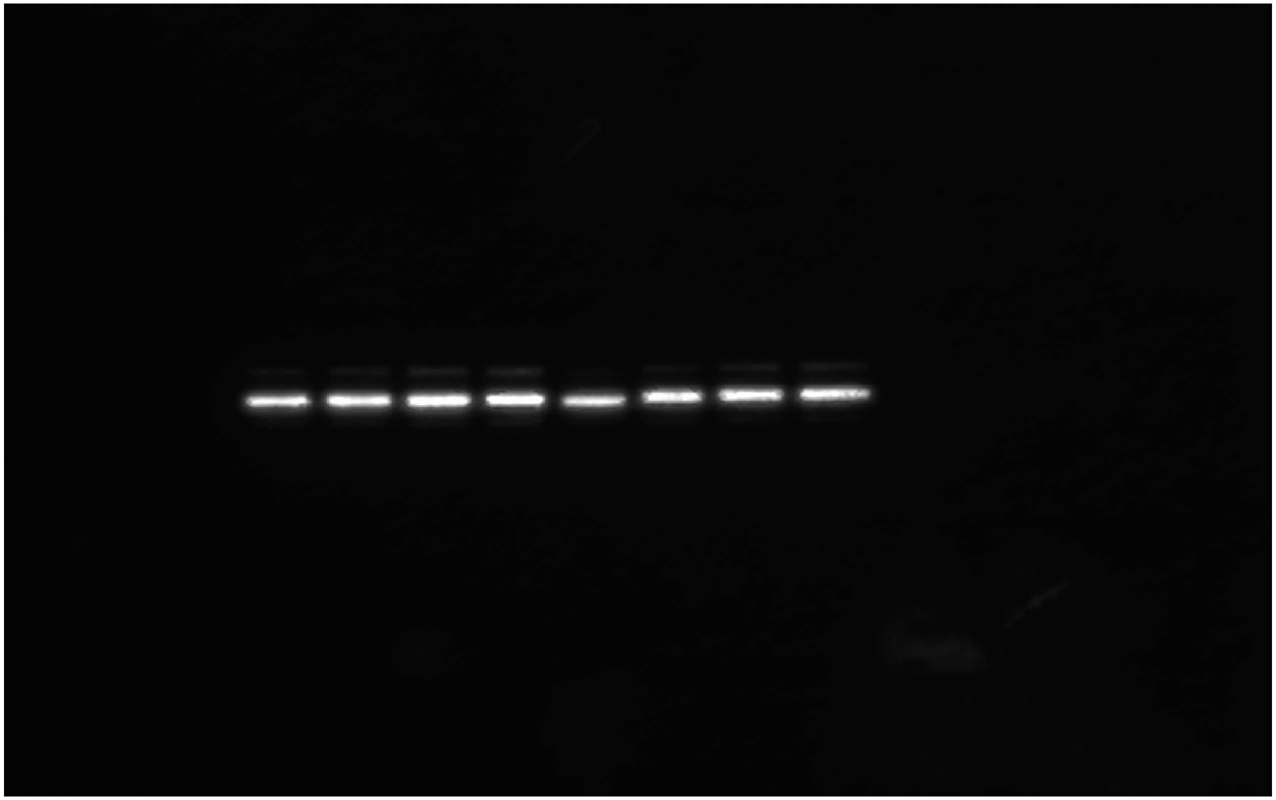

Fig. 7D
P70S6K

Western Blot raw image

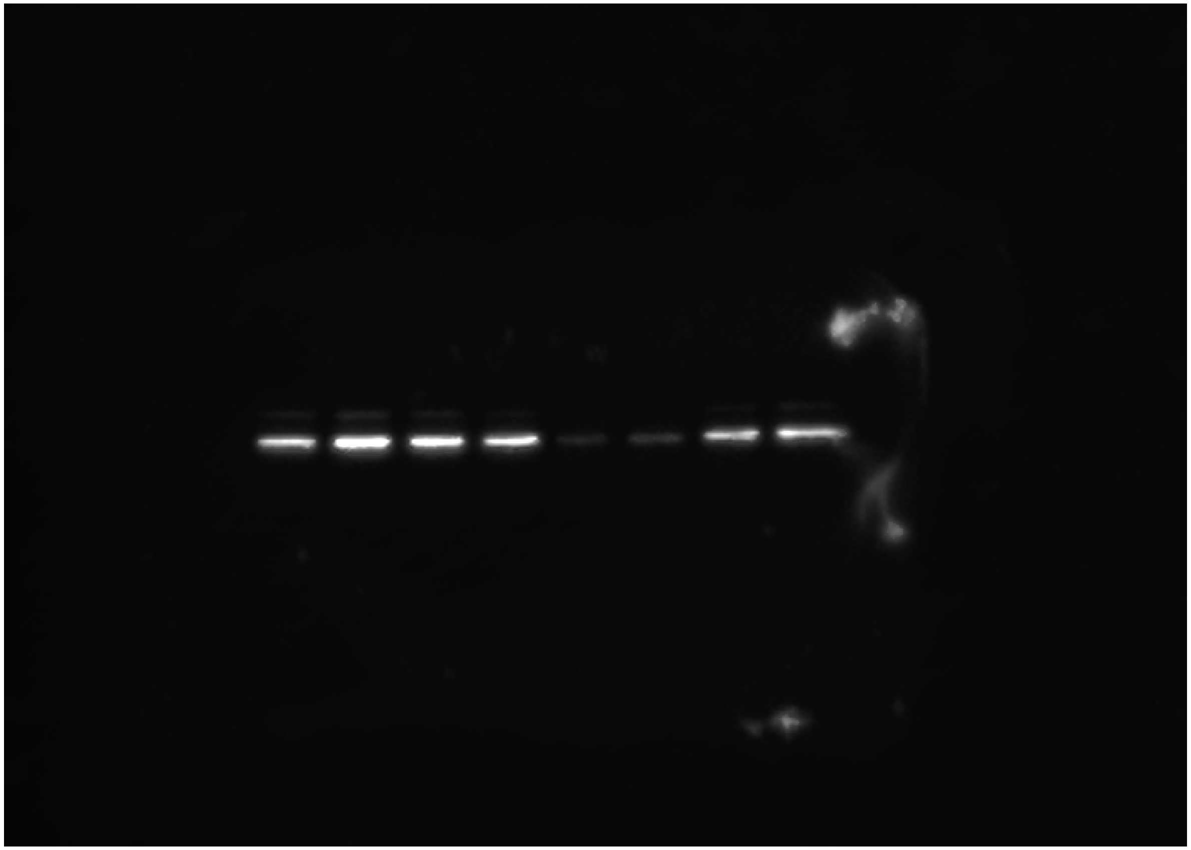

Fig. 7D
p-P70S6K

Western Blot raw image

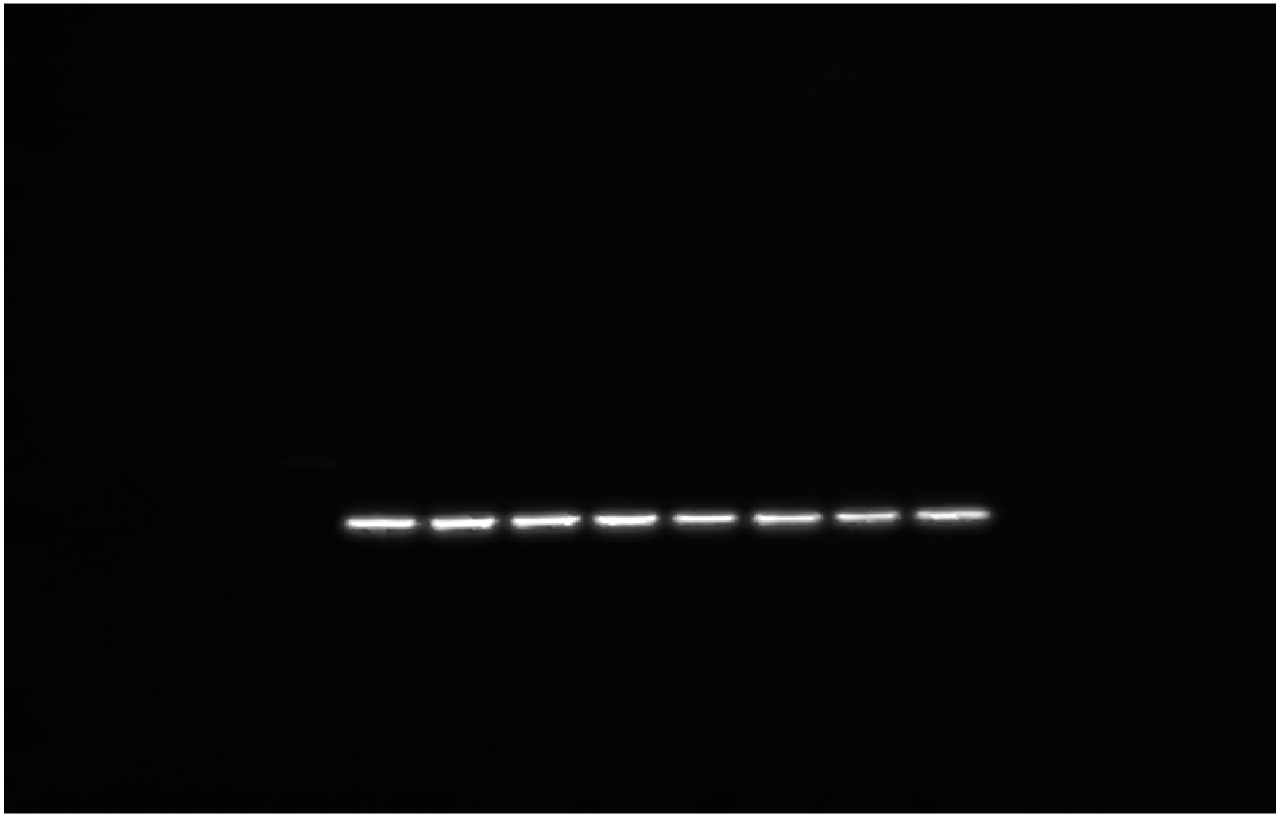

Fig. 7D
α-tubulin

Western Blot raw image

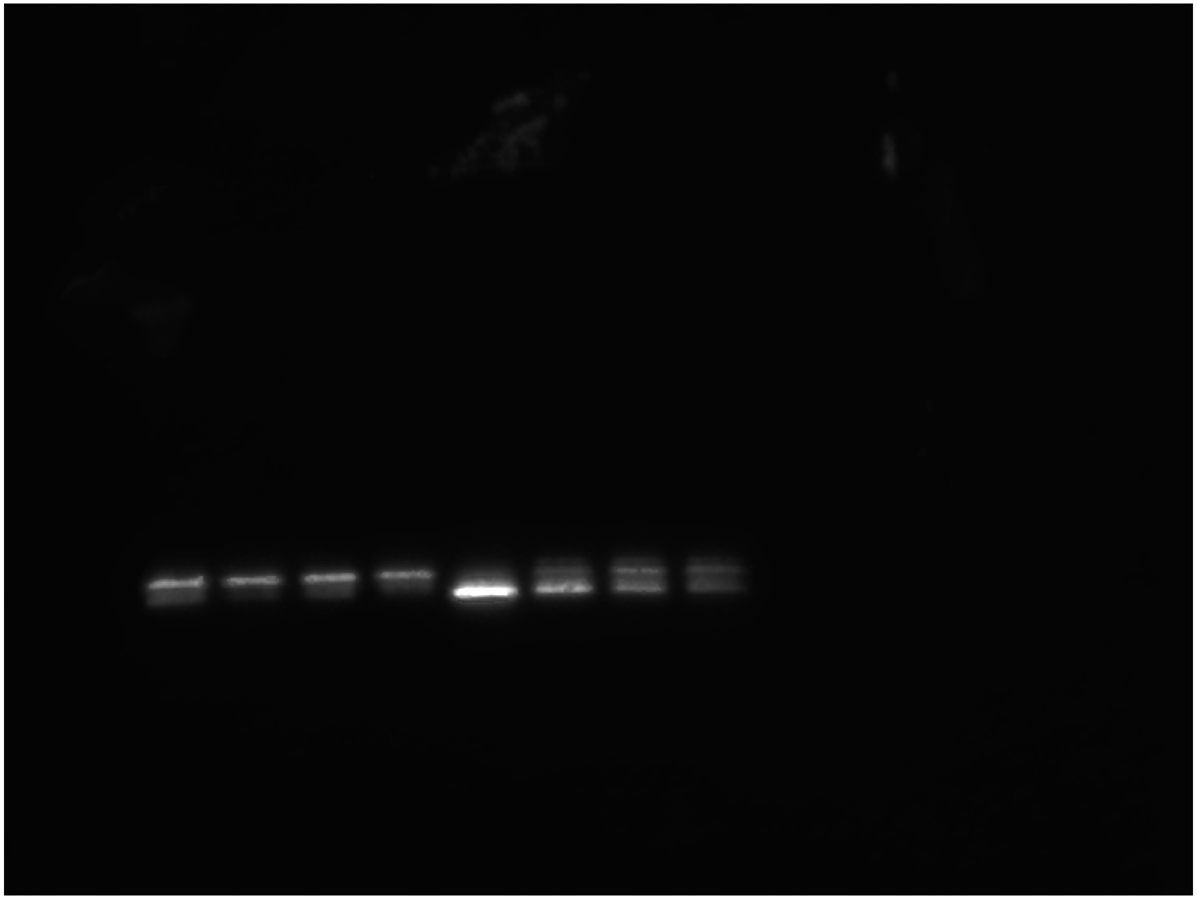

S1 Fig. A
4E-BP1

Western Blot raw image

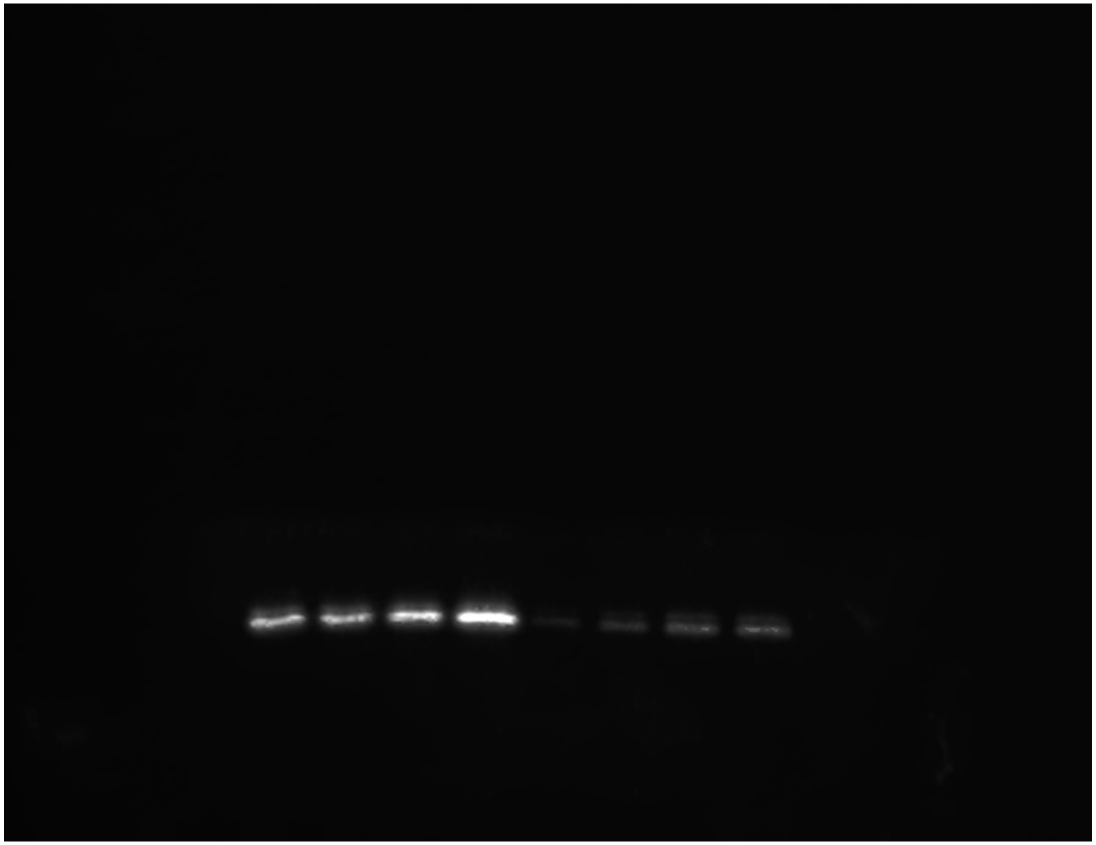

S1 Fig. A
P-4E-BP1

Western Blot raw image

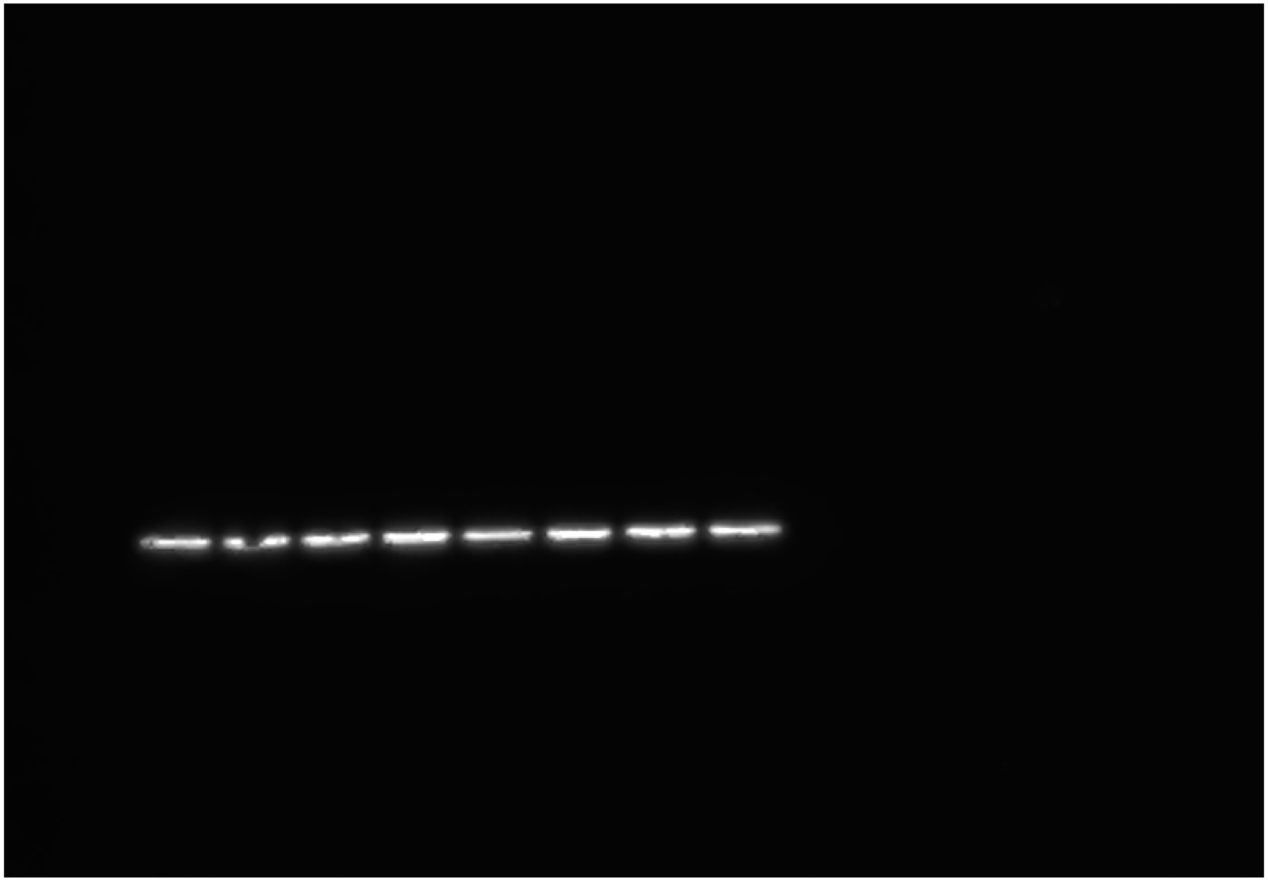

S1 Fig. A
α-tubulin

Western Blot raw image

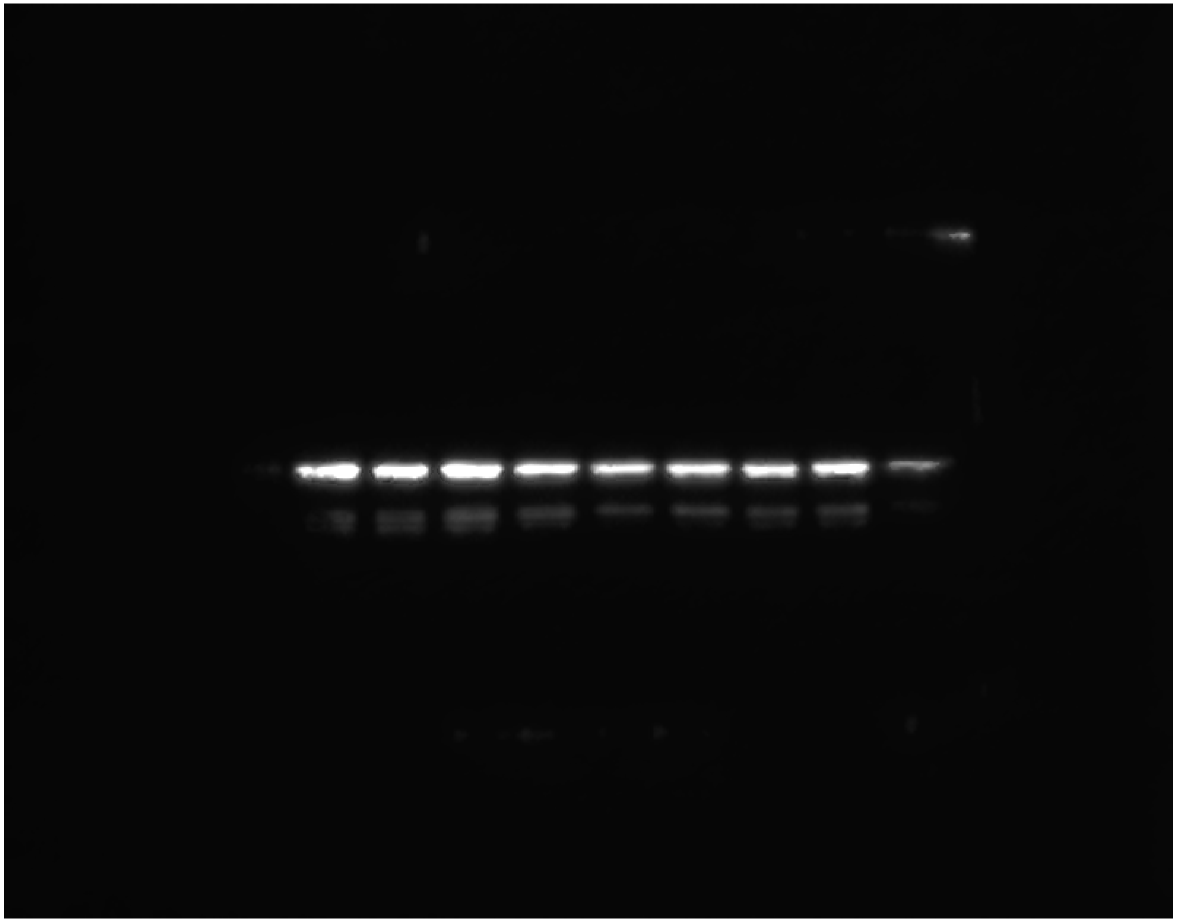

S1 Fig. D
SAPK/JNK

Western Blot raw image

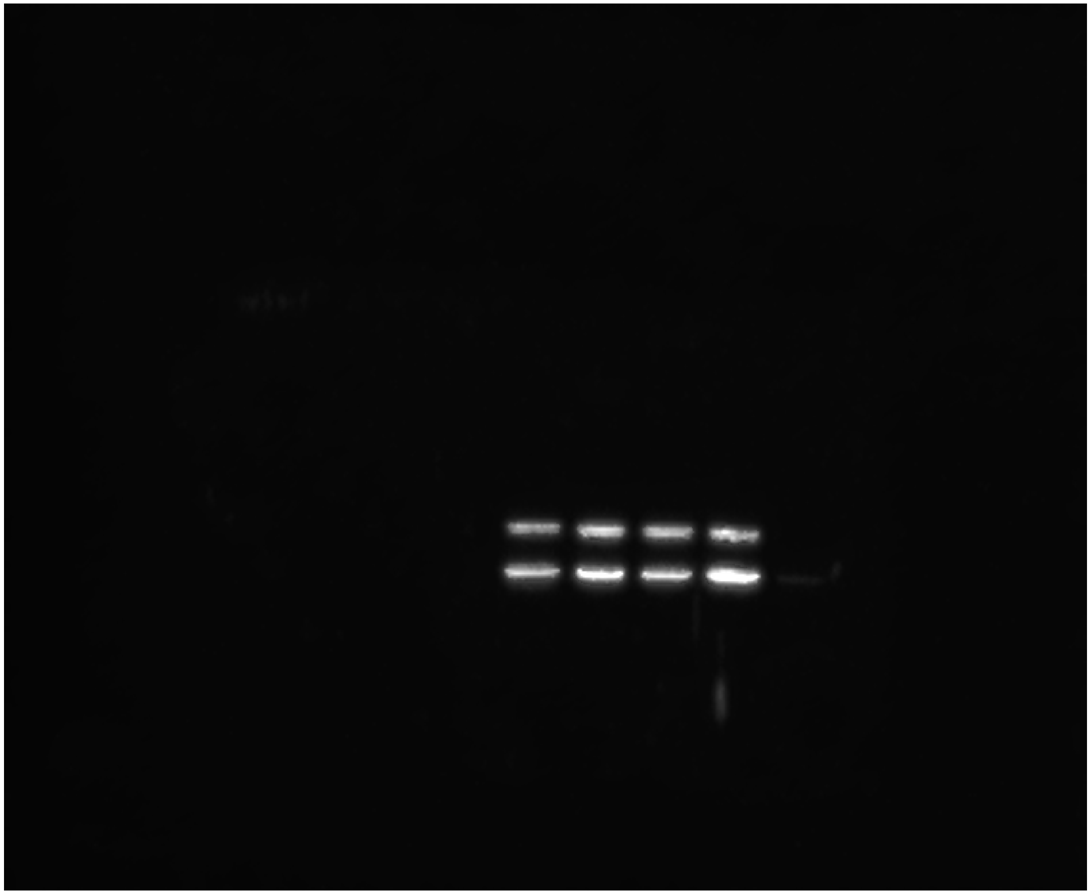

S1 Fig. D
P-SAPK/JNK

Western Blot raw image

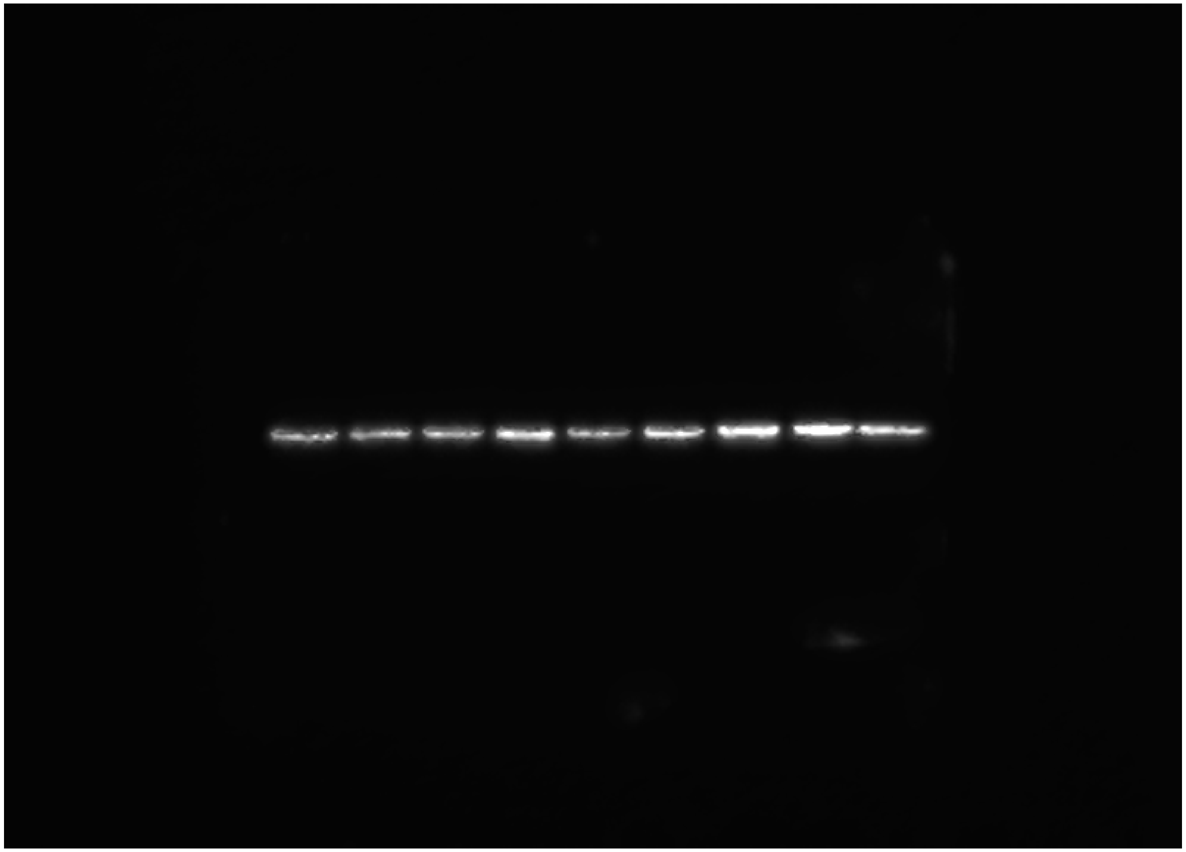

S1 Fig. D
α-tubulin
